# Author Correction: Puma genomes from North and South America provide insights into the genomic consequences of inbreeding

**DOI:** 10.1038/s41467-019-13096-3

**Published:** 2019-11-21

**Authors:** Nedda F. Saremi, Megan A. Supple, Ashley Byrne, James A. Cahill, Luiz Lehmann Coutinho, Love Dalén, Henrique V. Figueiró, Warren E. Johnson, Heather J. Milne, Stephen J. O’Brien, Brendan O’Connell, David P. Onorato, Seth P. D. Riley, Jeff A. Sikich, Daniel R. Stahler, Priscilla Marqui Schmidt Villela, Christopher Vollmers, Robert K. Wayne, Eduardo Eizirik, Russell B. Corbett-Detig, Richard E. Green, Christopher C. Wilmers, Beth Shapiro

**Affiliations:** 1 Department of Biomolecular Engineering, University of California, Santa Cruz, 1156 High Street, Santa Cruz, CA 95064 USA; 2 Department of Ecology and Evolutionary Biology, University of California, Santa Cruz, 1156 High Street, Santa Cruz, CA 95064 USA; 3 Department of Molecular, Cell, and Developmental Biology, University of California, Santa Cruz, 1156 High Street, Santa Cruz, CA 95064 USA; 4 Laboratório de Biotecnologia Animal, Departamento de Zootecnia, ESALQ, Universidade de São Paulo, Caixa Postal 09, Piracicaba, SP 13418-900 Brazil; 5 Department of Bioinformatics and Genetics, Swedish Museum of Natural History, P.O. Box 50007, Stockholm, 10405 Sweden; 6 Escola de Ciências, Pontifical Catholic University of Rio Grande do Sul, Avenida Ipiranga, 6681-Partenon, Porto Alegre-RS, 90619-900 Brazil; 7 Smithsonian Conservation Biology Institute, Smithsonian Institution, 600 Maryland Avenue SW, Washington, DC 20002 USA; 8 Theodosius Dobzhansky Center for Genome Bioinformatics, Saint Petersburg State University, 41 Sredniy Prospekt, Saint Petersburg, 199004 Russia; 9 Fish and Wildlife Research Institute, Florida Fish and Wildlife Conservation Commission, 298 Sabal Palm Road, Naples, FL 34114 USA; 10 Santa Monica Mountains National Recreation Area, 401 West Hillcrest Drive, Thousand Oaks, CA 91360 USA; 11 Department of Ecology and Evolutionary Biology, University of California, Los Angeles, 610 Charles E. Young Drive South, Los Angeles, CA 90095-1601 USA; 12 Yellowstone Center for Resources, P.O. Box 168, Yellowstone National Park, WY 82190 USA; 13 EcoMol Consultoria e Projetos, Avenida Limeira, 1131- Areiao, Piracicaba-SP, Brazil; 14 Environmental Studies Department, University of California, Santa Cruz, 1156 High Street, Santa Cruz, CA 95064 USA; 15 Howard Hughes Medical Institute, 400 Jones Bridge Road, Chevy Chase, MD 20815 USA; 16 Present Address: Laboratory of Neurogenetics of Language, Rockefeller University, 1230 York Avenue, New York, NY 10065 USA; 17 Present Address: Walter Reed Biosystematics Unit, Smithsonian Institution, 4210 Silver Hill Road, Suitland, MD 20746 USA; 18 Present Address: Department of Medical Informatics and Clinical Epidemiology, Oregon Health and Science University, 3181 S.W. Sam Jackson Park Road, Portland, OR 97239-3098 USA

**Keywords:** Computational biology and bioinformatics, Genomics, Conservation genomics, Population genetics, Conservation biology

Correction to: *Nature Communications* 10.1038/s41467-019-12741-1, published online 18 October 2019.

The original version of this Article contained several errors. In Figure 3a, both axes were incorrectly labelled as "PC2". The correct x axis label is "PC1". The present affiliation of Warren E Johnson with the Walter Reed Biosystematics Unit, Smithsonian Institution, was inadvertently omitted. The following statement was also omitted from the Acknowledgements: “Portions of this manuscript were prepared while W.E.J held a National Research Council Research Associateship Award at the Walter Reed Army Institute of Research and the published material reflects the views of the authors and should not be construed to represent those of the Department of the Army or the Department of Defense.” The initials “W.J” in the Author Contributions statement should have been listed as “W.E.J”. These errors have now been corrected in both the PDF and HTML versions of the Article.

