# Supplementary material for: Puma genomes from North and South America provide insights into the genomic consequences of inbreeding
Source: Nat Commun. 2019 Oct 18;10:4769. doi: 10.1038/s41467-019-12741-1 (PMC6800433; doi:10.1038/s41467-019-12741-1)
Supplement: Supplementary file 1 — Supplementary Information [file 41467_2019_12741_MOESM1_ESM.pdf]

Supplementary information

Puma genomes from North and South America provide insights into the genomic consequences of inbreeding

Nedda F. Saremi[+], Megan A. Supple[+], Ashley Byrne, James A. Cahill, Luiz Lehmann Coutinho, Love Dalén, Henrique V. Figueiró, Warren E. Johnson, Heather J. Milne, Stephen J. O'Brien, Brendan O'Connell, David P. Onorato, Seth P.D. Riley, Jeff A. Sikich, Daniel R. Stahler, Priscilla Marqui Schmidt Villela, Christopher Vollmers, Robert K. Wayne, Eduardo Eizirik, Russell B. Corbett-Detig, Richard E. Green, Christopher C. Wilmers, and Beth Shapiro[*]

[+] Contributed equally to this work
[*] Corresponding author beth.shapiro@gmail.com

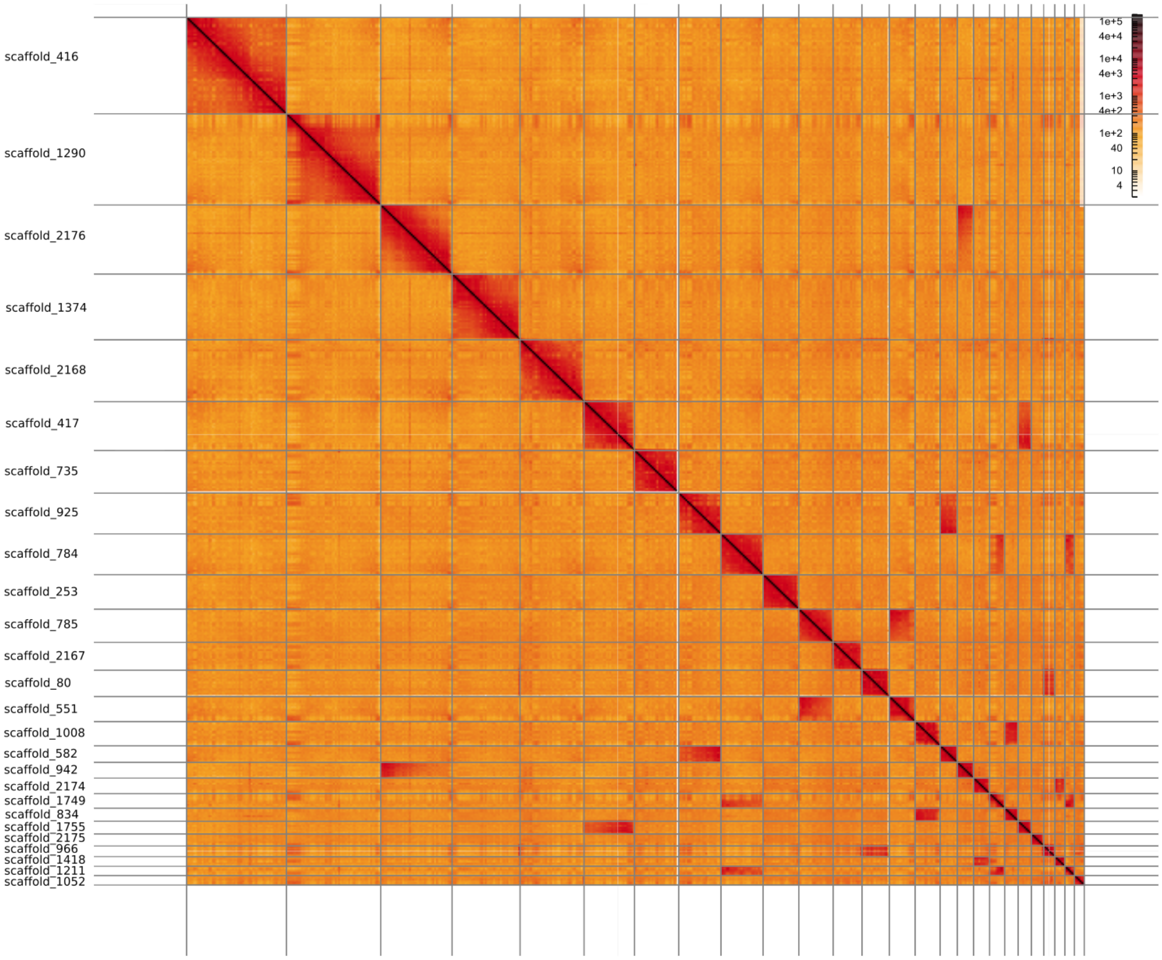

Supplementary Figure 1. Linkage map of the HiRise genome assembly for SC36. The x and y axes mark the mapping positions of the first and second read in a read pair of the Hi-C library, respectively. Mapping results are binned by genomic regions and each bin is colored according to the number of read pairs within the bin. Scaffolds less than 20 Mb in length are not shown.

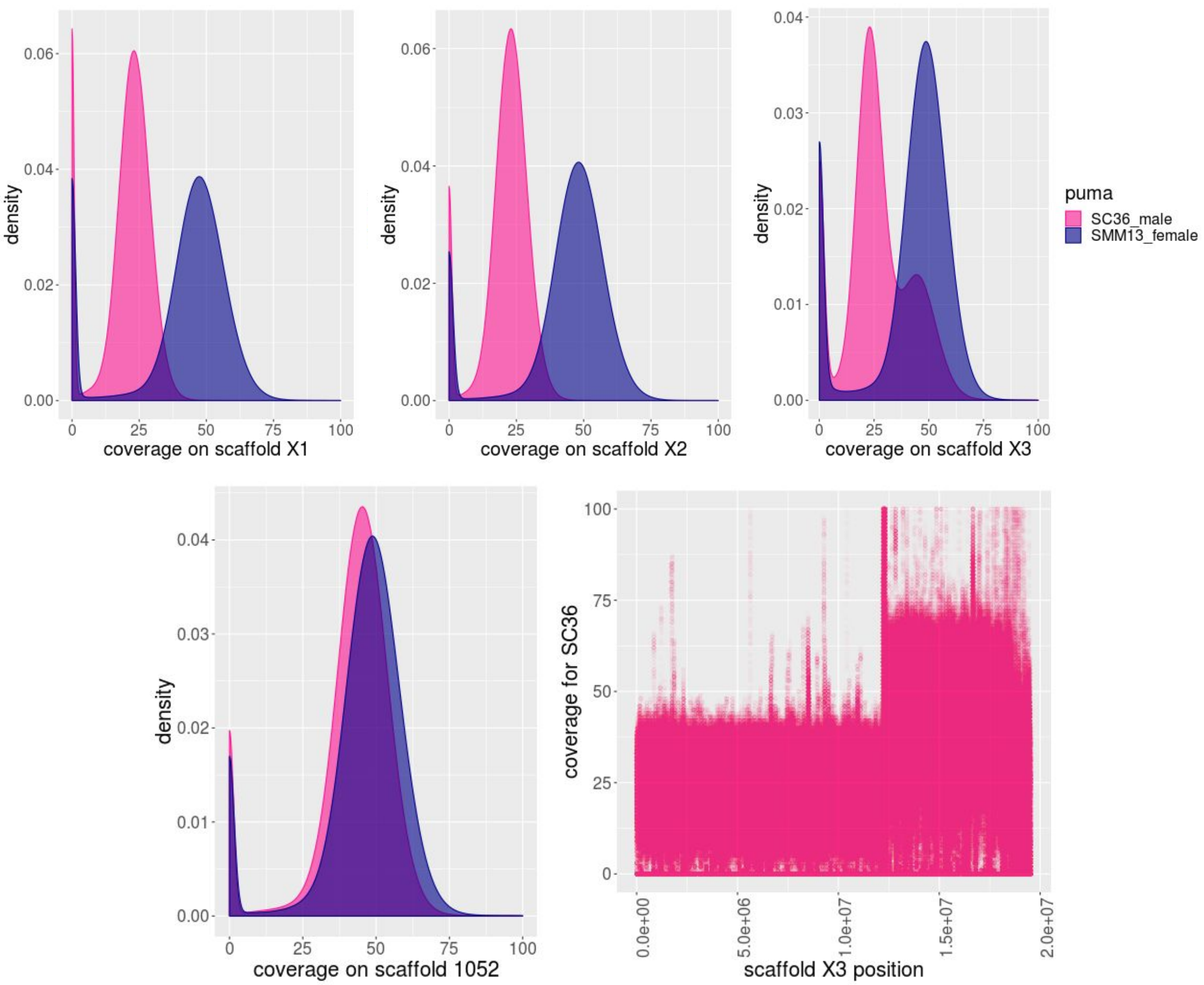

Supplementary Figure 2. Autosomal to X chromosome coverage ratio. Density coverage plots for the three X chromosome scaffolds (top) obtained from the SMM13 genome assembly and an autosomal scaffold (bottom left) from the SC36 genome assembly. Coverage is shown for SC36 (male) and SMM13 (female) mapped to the four scaffolds. For all X chromosome scaffolds, SC36 shows half the autosomal scaffold coverage. The trimodal distribution for SC36 on scaffold_X3 is due to the homology of pseudoautosomal region 1 between X and Y chromosomes resulting in twice the coverage for that region of the X in males. Coverage across scaffold_X3 for SC36 shows a doubling in average coverage at approximately the beginning of the pseudoautosomal region 1 (bottom right).

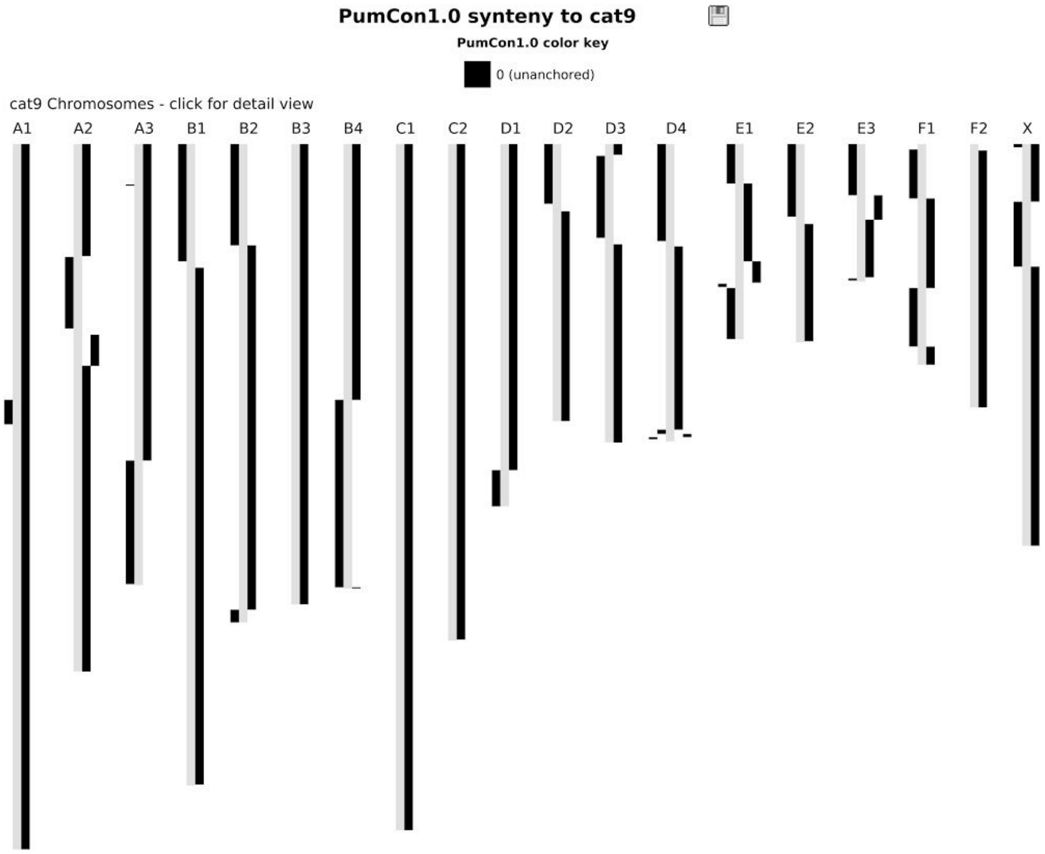

Supplementary Figure 3. Synteny between the puma and domestic cat genomes. Alignment of syntenic regions of the *Felis catus* genome version 9.0 (domestic cat, gray) with our PumCon1.0 assembly (black) using SyMAP[1].

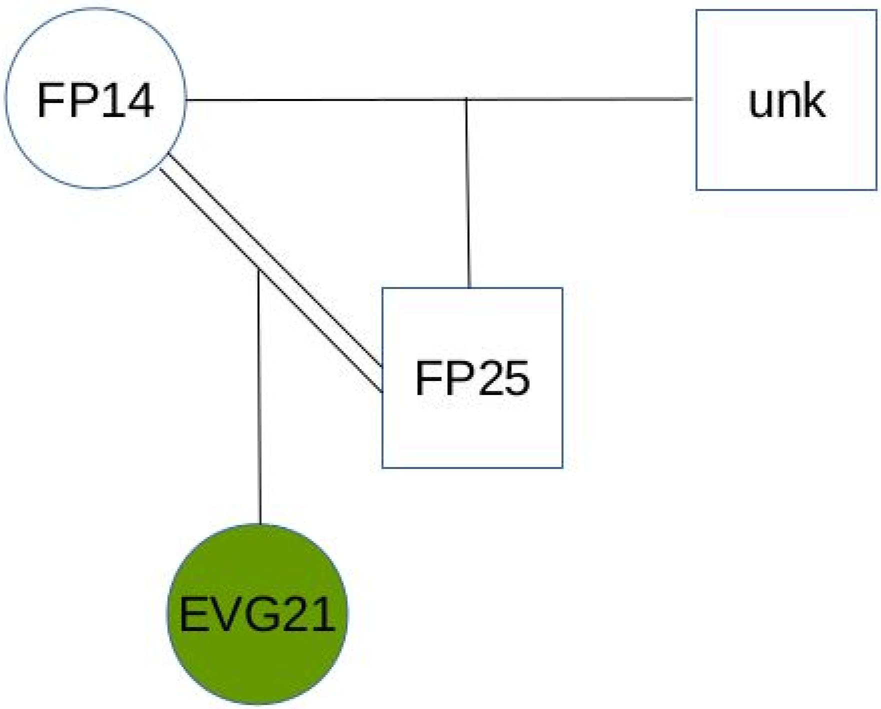

Supplementary Figure 4. Pedigree of EVG21. Pedigree of the inbred and admixed Florida panther sequenced from Everglades National Park[2]. All panthers in the pedigree are of Everglades ancestry. The Central American admixture into this population occurred approximately 6-9 generations prior to these individuals[2]. Note: EVG21 is referred to as FP021 in the original dataset[2].

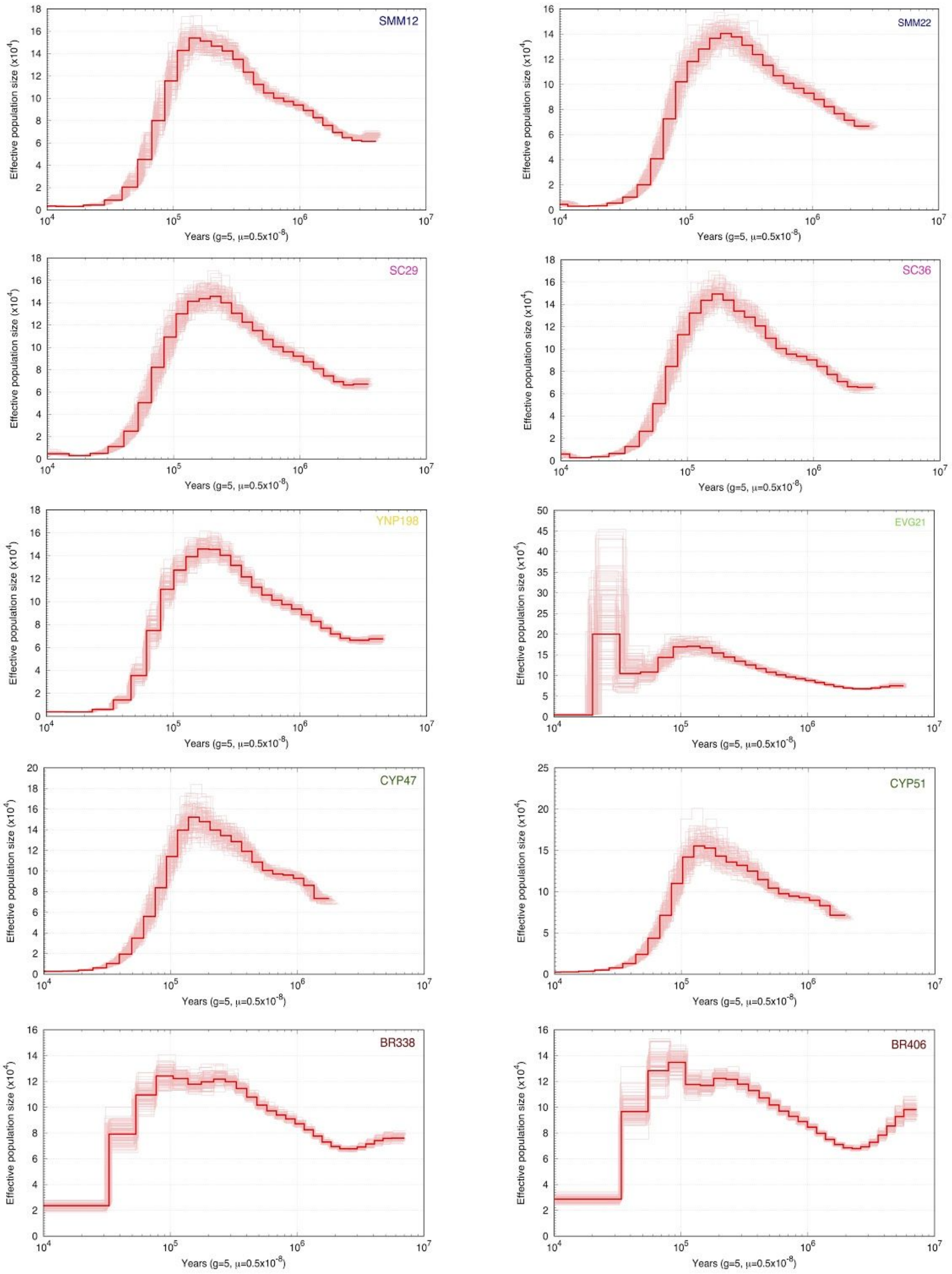

Supplementary Figure 5. Bootstrap replicate PSMC plots for ten pumas. We ran one hundred bootstrap replicates for each of the pumas using the PSMC model[3]. We used a generation time of 5 years, and a mutation rate of 0.5e-8[4] per bp per generation.

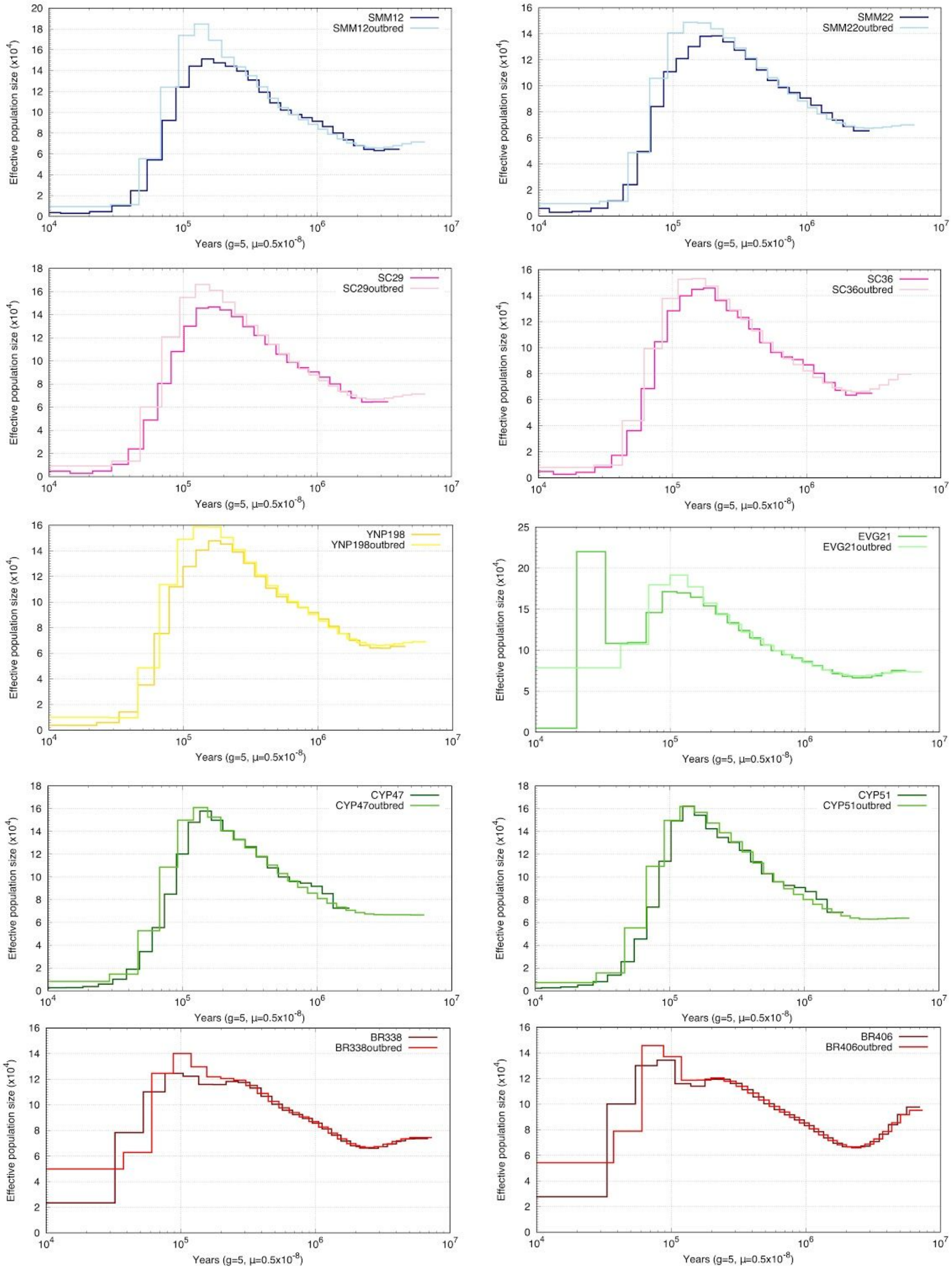

Supplementary Figure 6. PSMC plots for outbred (non-ROH) regions and entire genomes for the ten pumas. We see no substantial differences between the models[3]. We used a generation time of 5 years, and a mutation rate of 0.5e-8[4] per bp per generation.

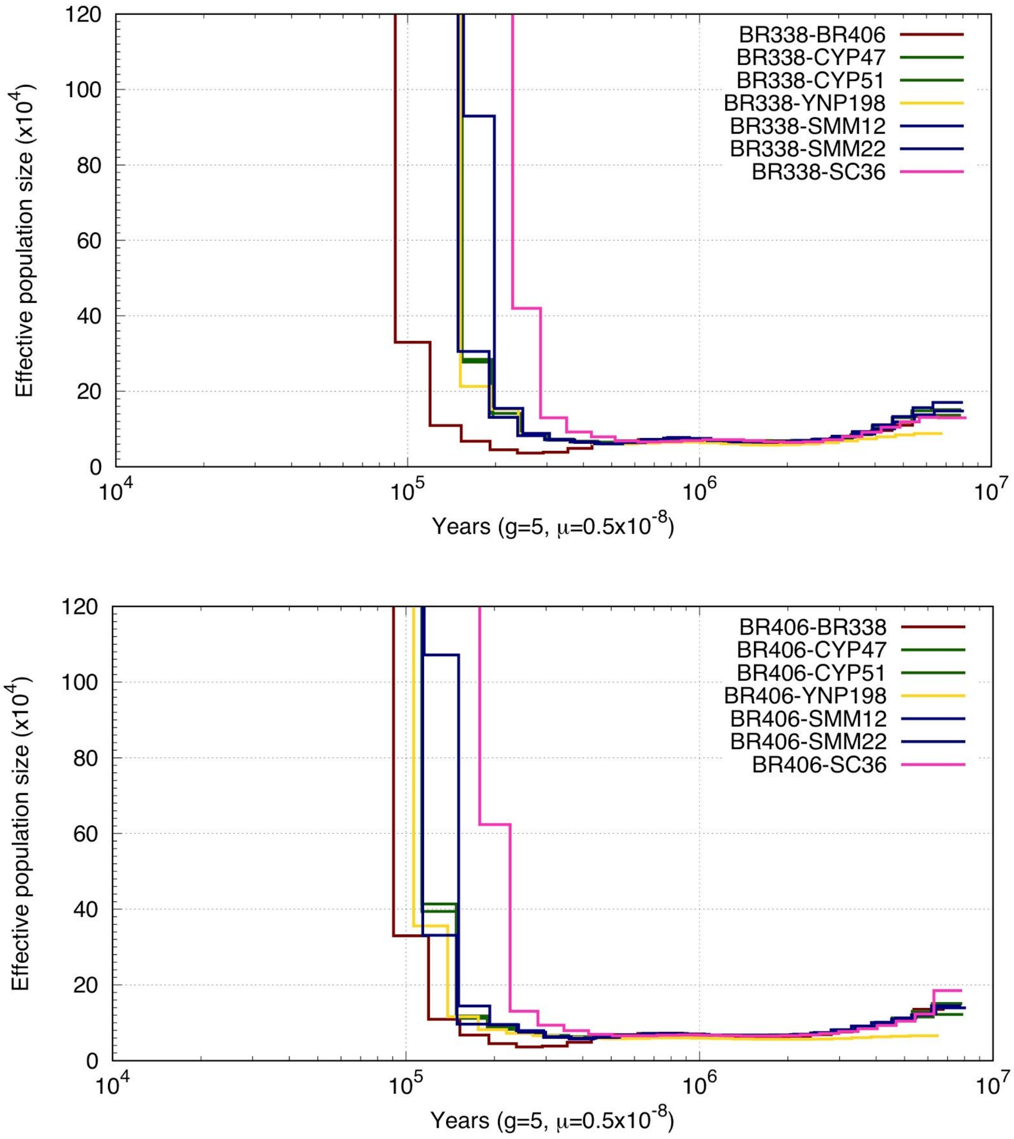

Supplementary Figure 7. Pseudo-diploid PSMC plots for X chromosomes of male pumas. We investigated the divergence times between our pumas by running PSMC modeling on pseudo-diploid sequences from pairs of haploid male X chromosomes. We see a sharp rise in the inferred N_e, and thus an approximate divergence time between 250-100 kya for every North American male when paired with a Brazil puma (either BR338 or BR406). The Brazil-Brazil pseudo-diploid pair had the most recent divergence time, roughly 100-90 kya. Thus the observed rise in effective population size observed in our autosomal PSMC model is not solely the result of population structure in pumas. We used a generation time of 5 years, a male to female mutation ratio of 1.4[5], and a mutation rate of 0.472e-8 per bp per generation.

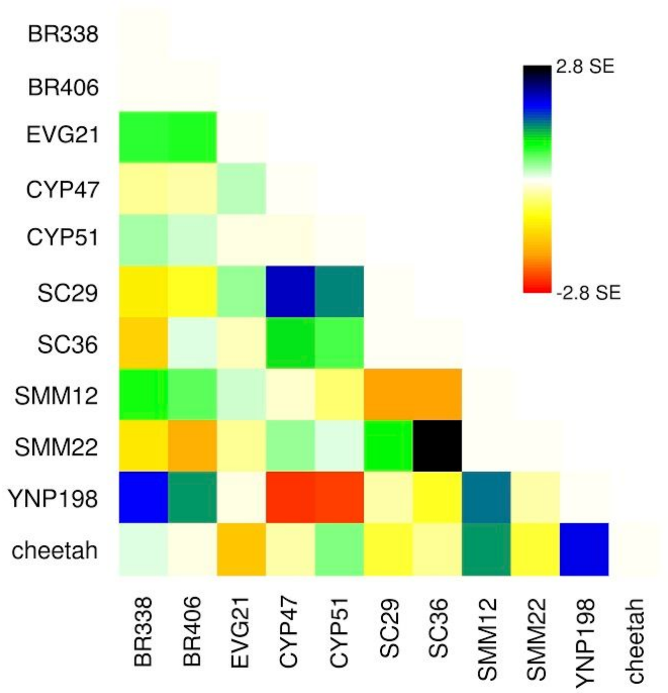

Supplementary Figure 8. TreeMix residual fit of the model. TreeMix[6] run on the LD filtered variant file containing ten pumas and the African cheetah, including one migration, and grouped SNPs together in windows of 5,000 SNPs (k). The model explained 99.91% of the variation.

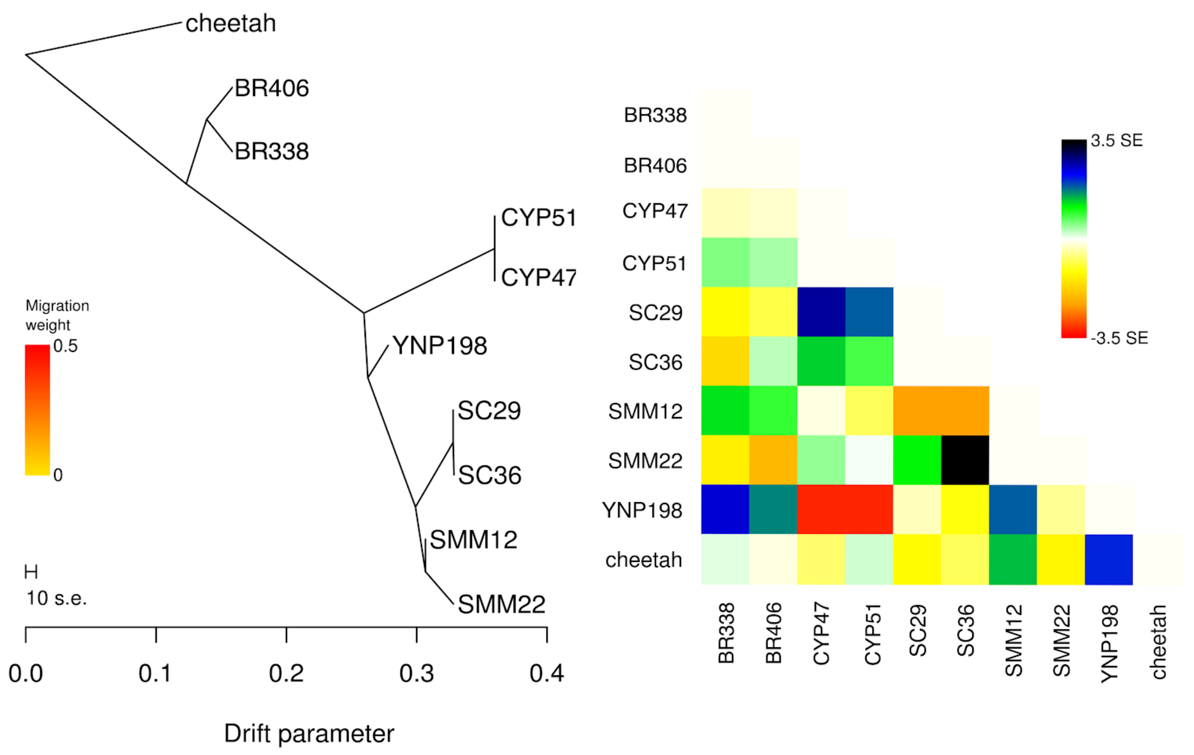

Supplementary Figure 9. TreeMix without EVG21. The result of TreeMix[6] with the highest likelihood when run on the LD filtered puma and cheetah variant file with EVG21 removed, including no migrations, and grouped SNPs together in windows of 5,000 SNPs (k). The model explained 99.91% of the variation.

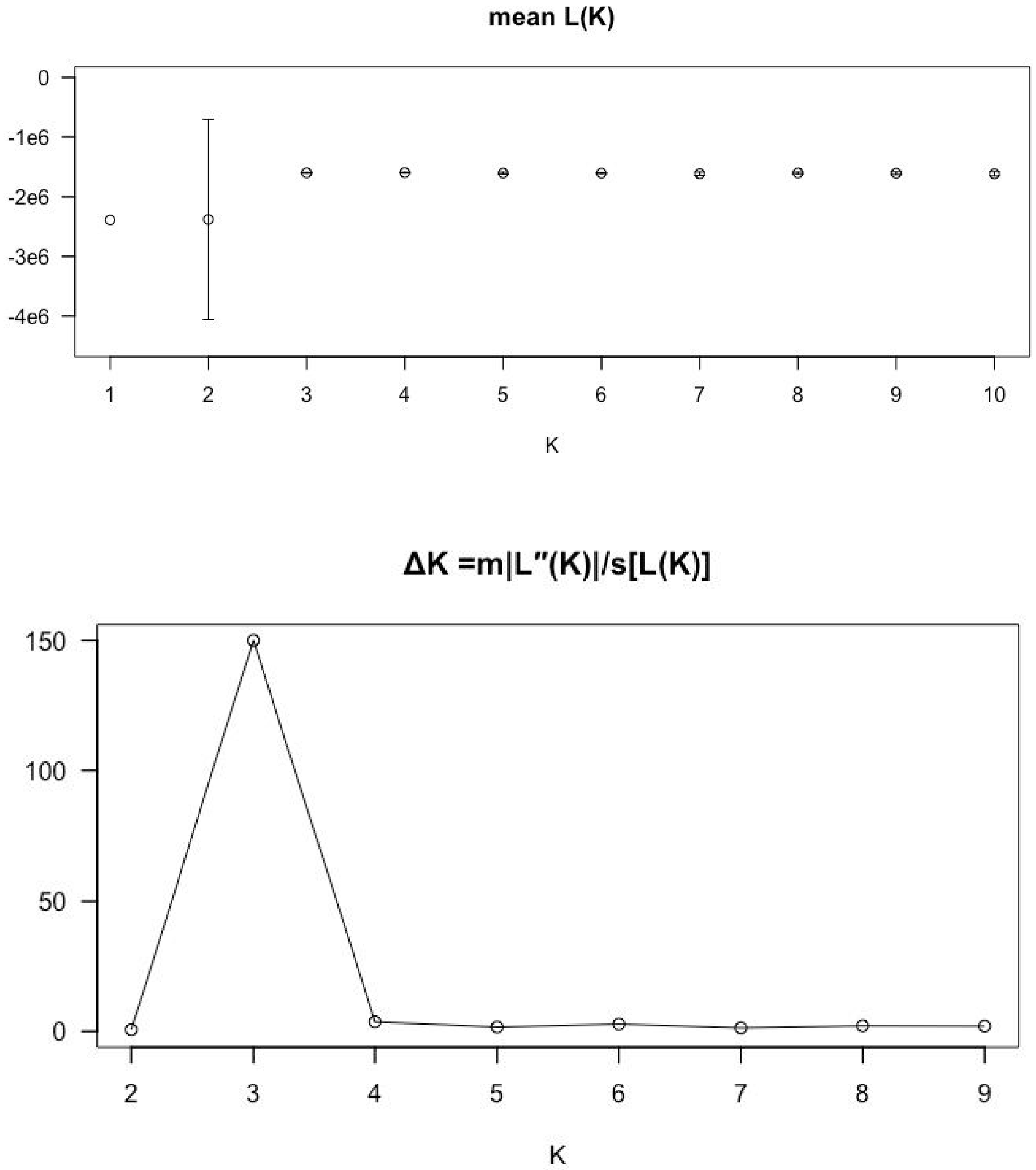

Supplementary Figure 10. Selection of best K in STRUCTURE. We identified K=3 as best for our panel using L(K) (top) and delta K (bottom)[7]. The top panel shows the mean likelihood (L(K)) and standard deviation from the 10 replicates per K value, which plateaued at K=3. The bottom panel shows that the rate of change in the log probability of data between successive K values (delta K) is highest at K=3. Raw data are provided as a Source Data file.

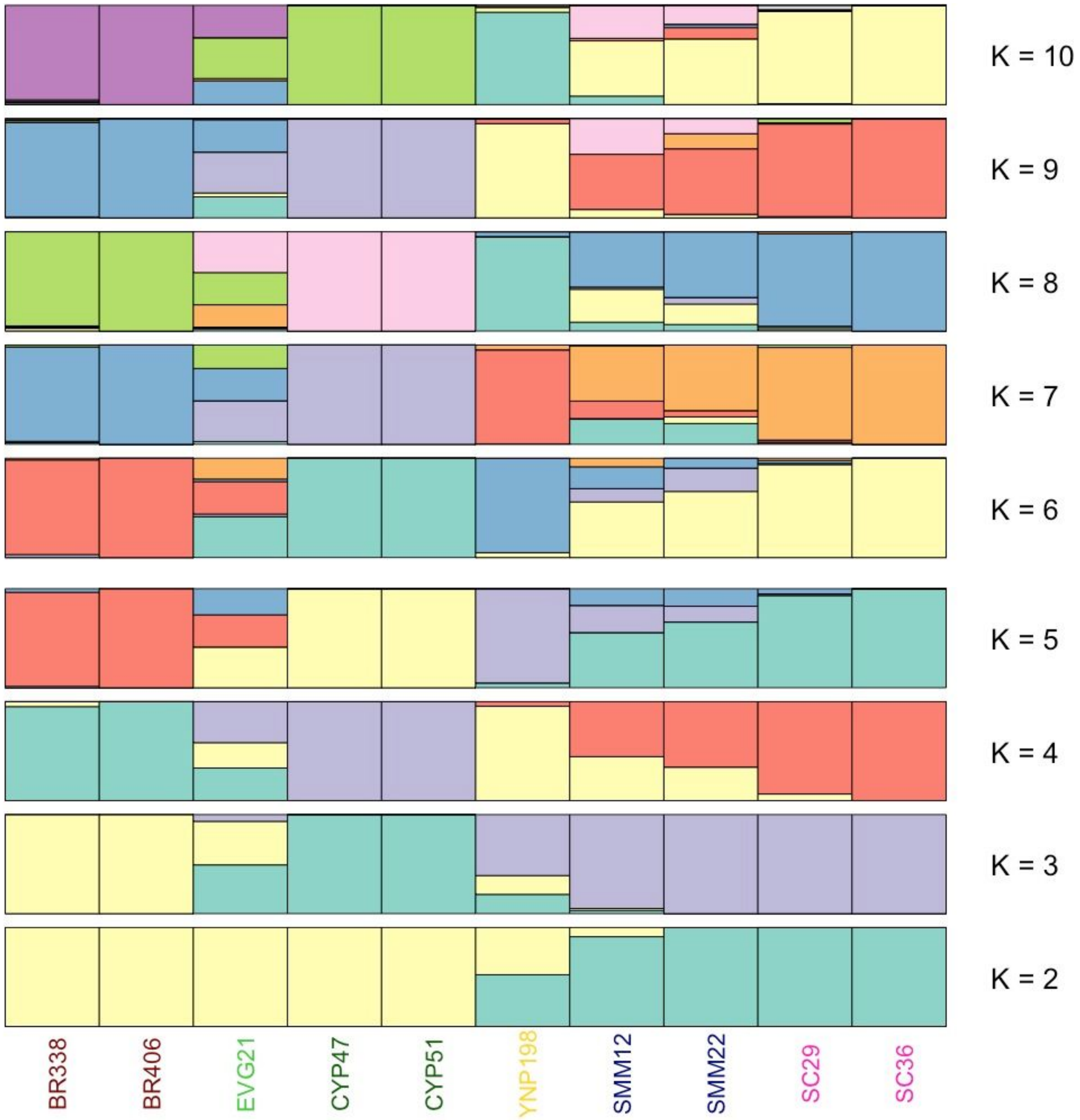

Supplementary Figure 11. STRUCTURE plots for K=2 through K=10. The mean of 10 permuted matrices of STRUCTURE[8] analysis for each of K=2 through K=10, performed using CLUMPP[9]. Both delta K and mean L(K) values indicated that K=3 was the best K for our panel (Supplementary Figure 10)[7].

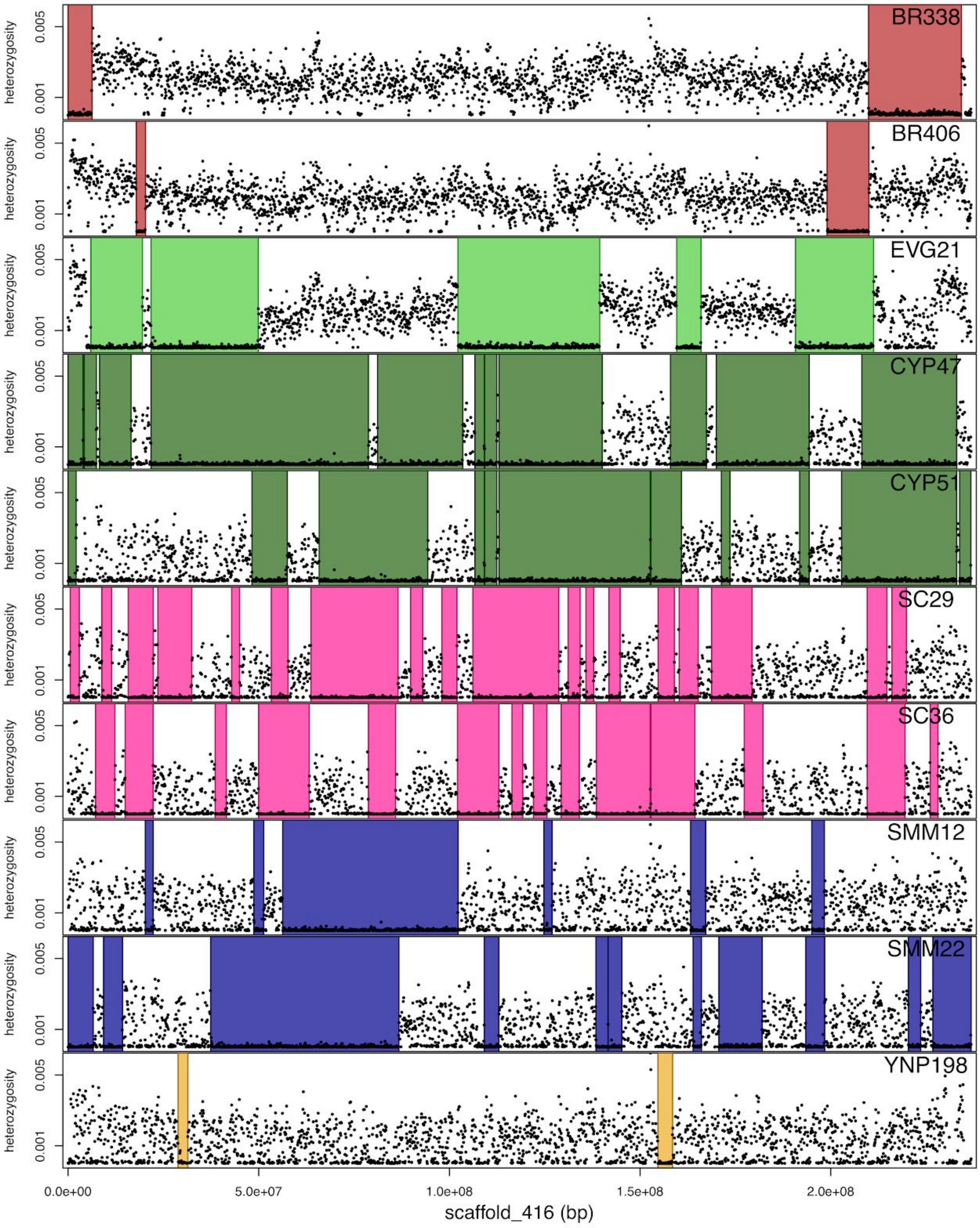

Supplementary Figure 12. ROH for all ten pumas as called by our ROH HMM. Black dots represent average heterozygosity in 100 kb windows; colored regions represent blocks called as ROH. Scaffold_416 represents the largest scaffold in the genome assembly at 236.8 Mb.

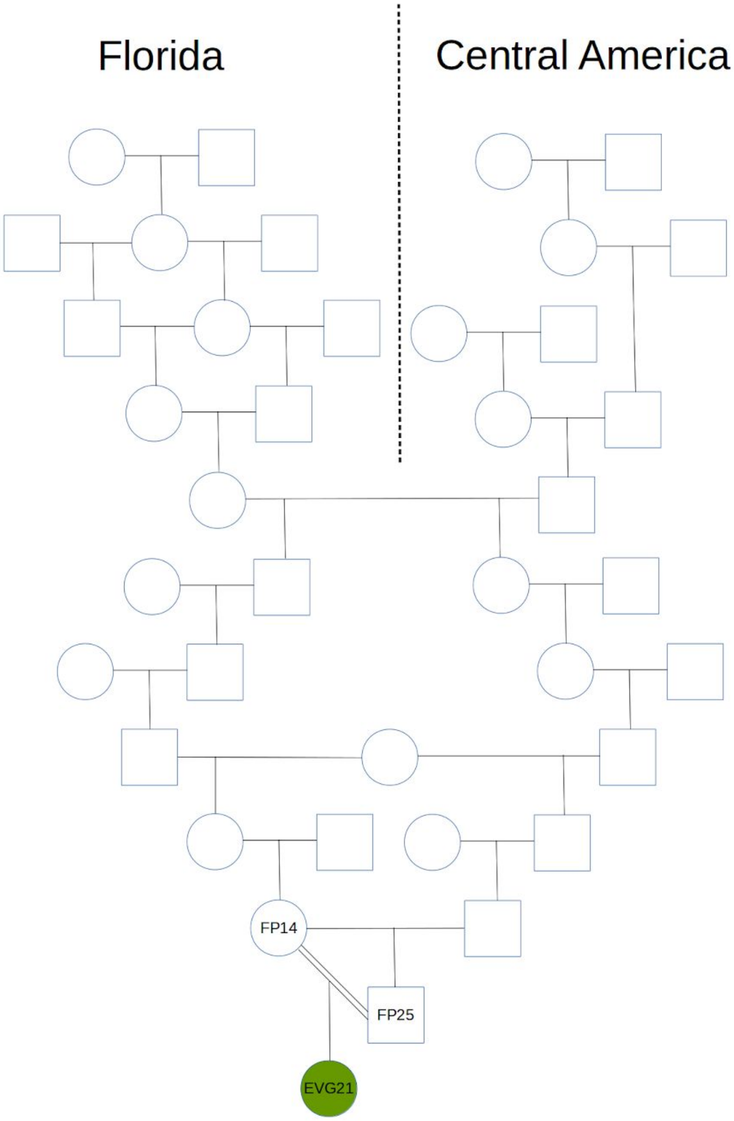

Supplementary Figure 13. Hypothetical pedigree for EVG21. Hypothetical pedigree showing how EVG21 could have both historical admixture and be the offspring of an inbreeding event. Early generations show separate Florida and Central American populations, which come together through admixture. The ancestral Floridian population was small and experienced considerable inbreeding. The ancestral Central American population would have been much larger and therefore experienced little inbreeding. The later generations show multiple inbreeding loops, where an individual's maternal and paternal ancestries trace back to a recent shared ancestor. Regions of the genome that are of mixed ancestry do not trace back to prior to the admixture event.

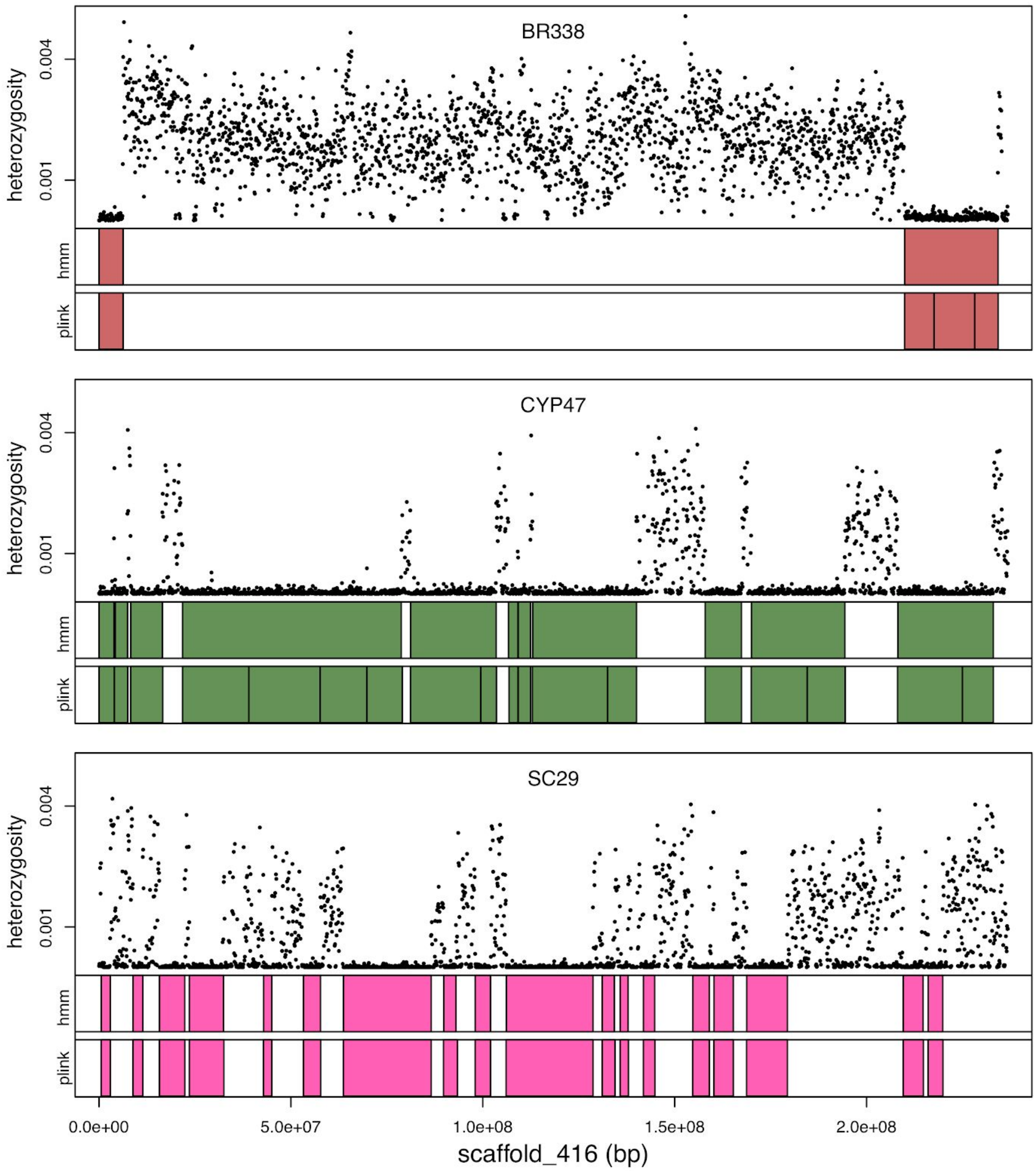

Supplementary Figure 14. ROH calls using two different methods. Top panel for each sample shows heterozygosity in 100 kbp windows. Bottom panels for each sample shows colored boxes indicating ROH called using our ROH HMM (top), and PLINK (bottom). PLINK tended to break up long tracts of ROH. Given that we were interested in the distribution of ROH lengths, we decided to use our HMM for ROH calls for further analyses.

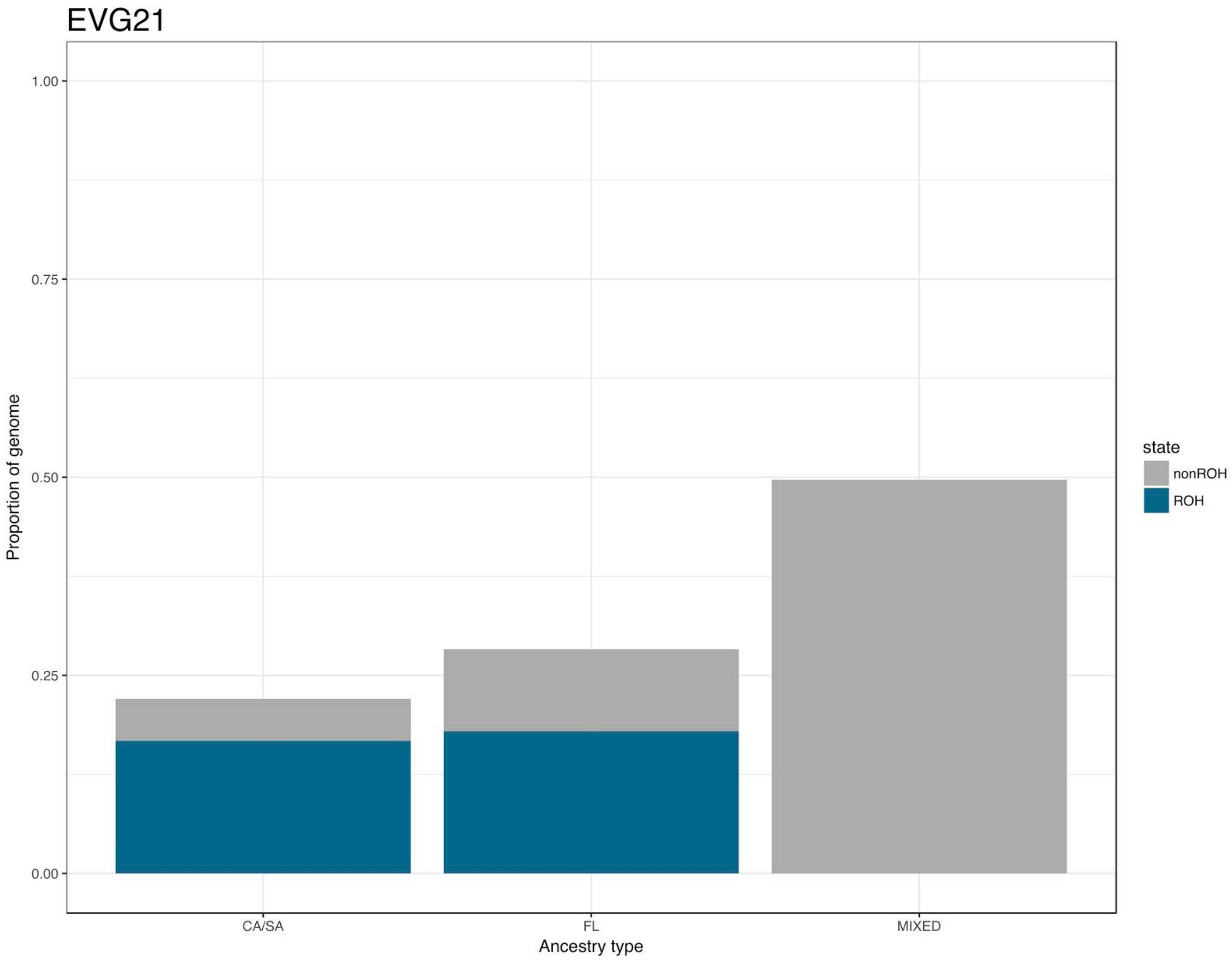

Supplementary Figure 15. Proportion of ancestry and ROH in genome of EVG21. The proportion of the genome classified as each of three ancestry types (pure Central/South American, pure Floridian, and mixed Central/South American and Floridian) by Ancestry_HMM[10,11]. The genome of EVG21 is composed of 21.98% Central/South American ancestry, 28.24% Floridian ancestry, and 49.58% mixed ancestry based on the HMM. For all ROH greater than 2 Mb, we determined the ancestry type as classified by the HMM. While we observe no ROH greater than 2 Mb in length in mixed ancestry regions for EVG21, we find numerous ROH greater than 2 Mb for both pure Central/South American ancestry and pure Floridian ancestry.

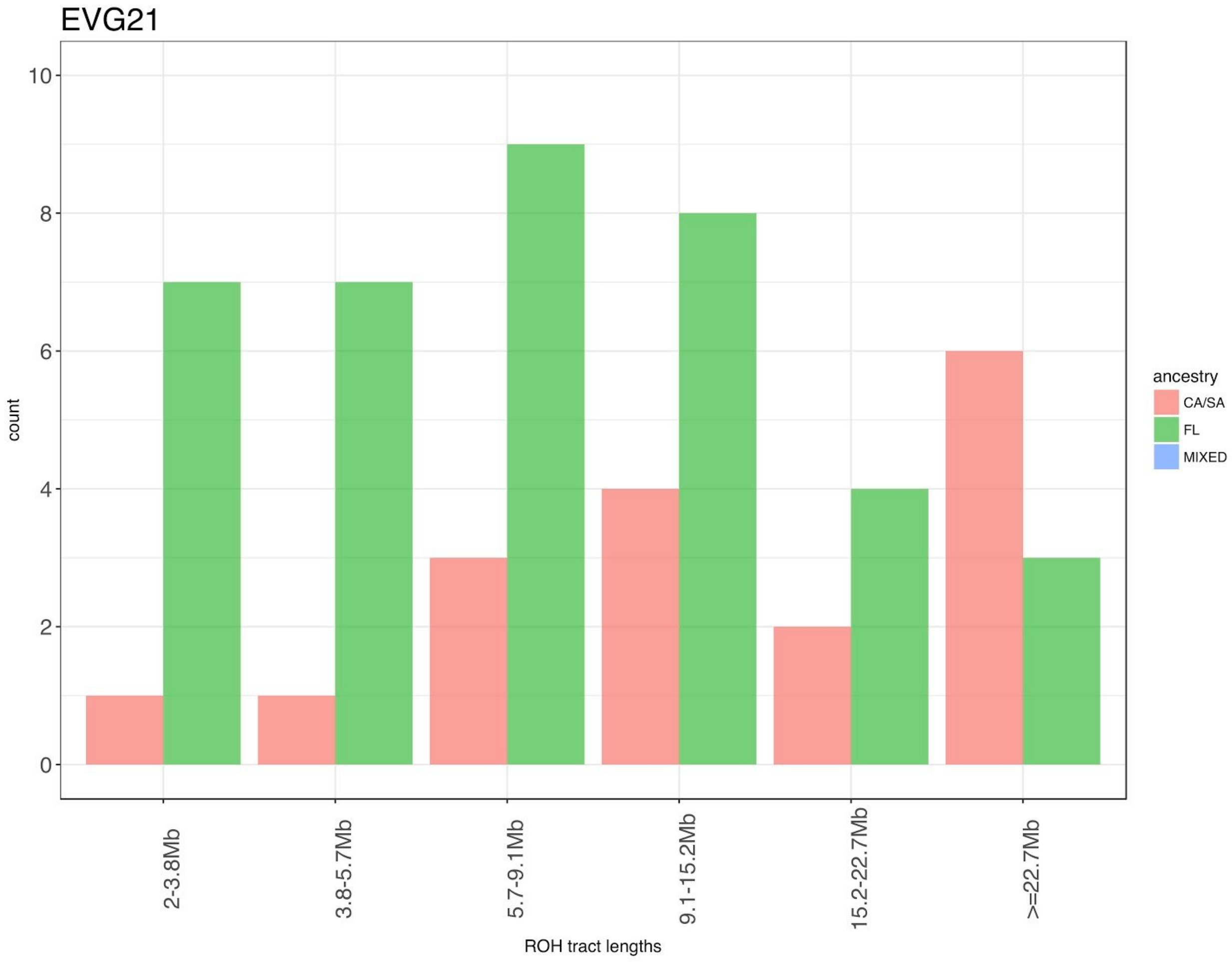

Supplementary Figure 16. Length distribution of ROH by ancestry type in EVG21 genome. Short ROH of Floridian ancestry occur at higher numbers than short ROH of Central/South American ancestry. This is likely due to a long history of small population size in Florida, resulting in inbreeding in the population prior to admixture.

Assembly version	Meraculous	HiRise	PBJelly	Pilon iteration 1	Pilon iteration 2
Assembly step	Shotgun assembly	Scaffolding	Gap filling	Error correcting	Error correcting
Input data	Illumina shotgun reads	Chicago & Hi-C libraries	ONT reads	Illumina shotgun reads	Illumina shotgun reads
Genome length (bp)	2,181,316,782	2,293,137,739	2,433,777,904	2,433,231,347	2,432,985,507
Contig N50	19.55kb	19.57kb	27.65kb	31.01kb	32.07kb
Scaffold N50	36.6kb	103.78 Mb	100.51 Mb	100.54 Mb	100.53 Mb
Scaffold L50	17,135	7	8	8	8
Scaffold NG50	35.1kb	103.78Mb	104.57Mb	104.60Mb	104.60Mb
Scaffold LG50	18,191	7	7	7	7
# of gaps	124,710	258,836	207,433	184,611	178,994
# of Ns	26,631,327	154,284,192	132,359,239	119,328,697	114,069,924
% of Ns in genome	1.22%	6.73%	5.44%	4.90%	4.69%
# Illumina shotgun reads mapping	958,095,130	979,540,843	982,339,501	985,819,801	987,305,346

Supplementary Table 1. Genome assembly metrics. The metrics of the puma genome at different stages of the assembly process. The final column represents the final assembly, PumCon1.0. We saw a marked improvement in the N50 as a result of scaffolding with HiRise. Gap filling with PB Jelly notably decreased the number of Ns and strings of Ns in the genome assembly. As a result of this conversion of Ns to useful sequence, the number of Illumina reads that mapped to the genome assembly increased. Due to the high error rate of ONT reads, the correction of the gap filled sequences using iterative rounds of Pilon with Illumina data also increased the number of reads that mapped to the genome. The Meraculous assembly did not include the final versions of the X chromosome scaffolds.

Complete BUSCOs	3832 (93.4%)
Complete and single-copy BUSCOs	3815 (93.0%)
Complete and duplicated BUSCOs	17 (0.4%)
Fragmented BUSCOs	141 (3.4%)
Missing BUSCOs	131 (3.2%)

Supplementary Table 2. Benchmarking Universal Single-Copy Orthologs (BUSCO) gene completeness score. The results of running BUSCO[12] on the PumCon1.0 genome using the human gene set (n=4104).

Puma	Population	Sex, Year sampled	Coverage	Alternate IDs	SRA accession IDs
BR338	Minas Gerais state, Brazil (BR)	male 2009	48X	bPco338[13]	SRR7639695-6
BR406	São Paulo state, Brazil (BR)	male 2013	27X	D406	SRR7542886-8
EVG21	Everglades National Park (EVG)	female 1987	51X	FP021, Pco-0075[2]	SRR7660678-9
CYP47	Big Cypress National Preserve (CYP)	male 1992	43X	FP047, Pco-0423[2]	SRR7664677-8
CYP51	Big Cypress National Preserve (CYP)	male 1992	55X	FP051, Pco-0428[2]	SRR7956993-4
YNP198	Yellowstone National Park (YNP)	male 2015	40X	M198	SRR7610940-1
SMM12	Santa Monica Mountains (SMM)	male 2009	46X	P12[14]	SRR7661934-5
SMM13*	Santa Monica Mountains (SMM)	female 2009	40X	P13[14]	SRR7690239-40
SMM22	Santa Monica Mountains (SMM)	male 2012	34X	P22[14]	SRR7543017-8
SC29	Santa Cruz Mountains (SC)	female 2014	35X	29F[15]	SRR7537344-5
SC36	Santa Cruz Mountains (SC)	male 2015	47X	36M[15]	SRR7148342-54

Supplementary Table 3. Details for the panel of pumas used in this study. *Note: SMM13 was used solely for the X chromosome scaffold assembly, and thus further metrics were not calculated.

Puma	Sex	Coverage	Heterozygosity (pileup method)	Heterozygosity (IUPAC filtered fastas)	Heterozygosity (GenomeScope, k=21)*	Proportion of genome in an ROH
BR338	male	48X	0.00155	0.00183	0.00352	0.06736
BR406	male	27X	0.00166	0.00183	0.00441	0.03970
EVG21	female	51X	0.00121	0.00128	0.00159	0.34221
CYP47	male	43X	0.00033	0.00037	0.00210	0.58485
CYP51	male	55X	0.00034	0.00040	0.00280	0.56168
YNP198	male	40X	0.00090	0.00096	0.00258	0.15873
SMM12	male	46X	0.00079	0.00086	0.00247	0.18884
SMM22	male	34X	0.00059	0.00059	0.00249	0.417637
SC29	female	35X	0.00062	0.00067	0.00165	0.32707
SC36	male	47X	0.00049	0.00060	0.00270	0.33987

Supplementary Table 4. Coverage and heterozygosity values for the panel of pumas used in this study. *Note: For samples with lower coverage and/or lower genome-wide heterozygosity, the two peaks used by GenomeScope to estimate heterozygosity were not clearly discernible, reducing the accuracy of the estimates. Additionally, we expect that males and females will show differences due to having heterogametic or homogametic sex chromosomes for the GenomeScope values.

Sample	Genotyping error rate	Outbred heterozygosity
BR338	0.000146	0.0019
BR406	0.000054	0.0018
EVG21	0.000071	0.0019
CYP47	0.000078	0.0011
CYP51	0.000071	0.0010
SC29	0.000078	0.0012
SC36	0.000027	0.0010
SMM12	0.000075	0.0012
SMM22	0.000078	0.0012
YNP198	0.000087	0.0012

Supplementary Table 5. ROH HMM parameters. Parameters used as input into the HMM ROH script for each puma.

	BR338	BR406	EVG21	CYP47	CYP51	SC29	SC36	SMM12	SMM22	YNP198
BR338	6.7									
BR406	0.0	4.0								
EVG21	0.0	0.0	34.2							
CYP47	0.0	0.0	8.7	58.5						
CYP51	0.0	0.0	6.8	35.9	56.2					
SC29	0.0	0.0	0.2	1.2	1.1	32.7				
SC36	0.0	0.0	0.1	0.7	0.7	11.6	34.0			
SMM12	0.0	0.0	0.0	0.6	0.5	3.0	2.7	18.9		
SMM22	0.0	0.0	0.1	0.9	0.8	5.1	4.1	4.4	41.8	
YNP198	0.0	0.0	0.2	0.3	0.2	0.6	0.2	0.7	0.6	15.9

Supplementary Table 6. Pairwise ROH IBD values. Percent of the genome in an IBD ROH between pairs of pumas as shown in Fig. 4D. Diagonal values are the percentage of the genome in ROH for each individual.

Supplemental Methods

Assembly and annotation of the puma reference genome

We performed two DNA extractions using the Qiagen DNeasy Blood & Tissue kit following the manufacturer's protocol for non-nucleated erythrocytes using 100 μL of blood. We made four indexed Illumina libraries from these two extractions, following the Meyer Kircher protocol[16], targeting insert sizes of 350bp and 550bp. We created a third DNA extraction for SC36 using the Qiagen Blood and Cell Culture Mini Kit using 500 μL of blood, following the manufacturer's protocol up until the spooling step. The DNA was not concentrated enough to precipitate, so instead we centrifuged the sample, washed it with cold 70% EtOH, and further centrifuged it. We removed the supernatant and air dried the pellet. We resuspended the pellet in 50 μL of TE buffer at 55°C for 2 hours. We prepared four indexed Illumina libraries from this extraction following the Meyer Kircher protocol[16], targeting an insert size of 330bp. We sent out the eight paired-end shotgun libraries for sequencing at UC Berkeley Vincent J. Coates Genomics Sequencing Laboratory on an Illumina HiSeq 2500 (2x150bp, 2x100bp), at UC Santa Cruz Ancient and Degraded Processing Center on an Illumina MiSeq (v3 chemistry, 2x300bp), and at UC San Diego Institute for Genomic Medicine Genomics Center on an Illumina HiSeq 2500 (2x100bp).

We extracted additional DNA from the SC36 blood samples using the Qiagen Blood and Cell Midi Kit (catalog no. 13343). We quantified DNA using a Qubit fluorometer. We assembled 1μg of in vitro chromatin using the Active Motif In Vitro Chromatin Assembly kit, according to the manufacturer's instructions. We crosslinked the chromatin in 1% formaldehyde for 15 minutes at room temperature, and quenched with 2.5M Glycine. We immobilized chromatin on SPRI beads at a SPRI-lysate ratio of 2:1[17], and washed with 10mM Tris and 50mM NaCl to remove non-histone-associated DNA. We resuspended the bead and chromatin mixture in 49.5μL 1X NEBuffer 2, and digested with 5 units of DpnII enzyme for one hour at 37°C in a thermal-mixer. After digesting, we concentrated the beads and washed twice with the wash buffer. We resuspended the sample in a 50 μL reaction containing dA-dT-and dGTP, biotinylated dCTP, and Klenow. We performed end-labeling at 25°C for 30 minutes, after which we washed the sample twice with the wash buffer. The sample was ligated overnight in a 250 μL reaction containing 1X NEB T4 ligase buffer, 0.1mg per ml BSA, 0.25% Triton X-100, and 50 units T4 DNA ligase. After ligation, we added 2.5μL 10mM dNTPs and 5.5 units T4 DNA polymerase to remove unligated biotin-dCTP. After concentrating the sample and removing the ligation buffer, the crosslinks were reversed and the sample was deproteinated in 50 μL cross-link reversal buffer (50mM Tris pH=8.0, 1% SDS, 0.25mM CaCl2, and 0.5 mg per mL Proteinase K). We incubated the sample at 55°C to digest the histones, then increased the temperature to 68°C to reverse crosslinks. We then separated the sample from the beads and purified on fresh SPRI beads at a ratio of 2:1[18]. DNA recovery was quantified with a Qubit fluorometer. We prepared 400ng of sample for sequencing using the NEB Ultra library preparation kit according to the manufacturer's instructions, with one exception: prior to the indexing PCR, the sample was enriched by pulldown on 30 μL Invitrogen C1 Streptavidin beads, then washed to remove non-biotinylated DNA fragments. The washes were as follows: 1

wash with lithium wash buffer (1M LiCl, 0.5mM EDTA, 0.05% Tween-20), followed by 2 washes with sodium wash buffer (1M NaCl, 0.5mM EDTA, 0.05% Tween-20), and 2 washes with TE-Tween. We sent the library for sequencing at the UC San Diego Institute for Genomic Medicine Genomics Center on an Illumina HiSeq 2500[19] (2x125bp reads).

We generated a Hi-C library for SC36 by centrifuging 1mL of blood stored in EDTA at 2500 rcf for 5 minutes to pellet and washing with 1mL PBST, then resuspending in 100ul PBST. We crosslinked the cells with 1% final concentration formaldehyde for 15 minutes at room temperature and quenched the reaction with 2.8μL 2.5M Glycine, and pelleted to exchange the buffer. We incubated the cells in 500μL lysis buffer (10mM HEPES pH-8.0, 10mM NaCl, 0.2% IGEPAL CA-630, and 1X Protease inhibitors solution (Roche)), and then lysed them further with the addition of 0.5mm garnet beads (MoBio PowerMax beads) by adding 100μL of beads and vortexing the tube on its side for 1 minute at max speed. We decanted the lysate, and then purified the chromatin by centrifuging at 2500 rcf for 5 minutes, followed by 3 washes with 200μL wash buffer (10mM Tris ph=7.5, 50mM NaCl, 0.1% Tween-20) before resuspension in 55μL wash buffer with 1μL 2% SDS, followed by incubation at 37°C for 20 minutes[20]. We made two technical replicates with 1μg of chromatin per replicate, as determined by Qubit fluorometry. We immobilized chromatin on SPRI beads at a SPRI-lysate ration of 2:1[17], then washed with 10mM Tris, 50mM NaCl to remove non-histone-associated DNA. We resuspended the bead/chromatin mixture in 49.5μL 1X NEBuffer 2, and digested with 5 units of DpnII enzyme for one hour at 37°C in a thermal-mixer. After digesting, we concentrated the beads and washed them twice with the wash buffer. We resuspended the sample in a 50 μL reaction containing dA-dT-and dGTP, biotinylated dCTP, and Klenow. We performed end-labeling at 25°C for 30 minutes, then washed the sample twice with the wash buffer. We ligated the sample overnight in a 200 μL reaction containing 1X NEB T4 ligase buffer, 0.1mg per ml BSA, 0.25% Triton X-100, 1mM DTT, and 50 units T4 DNA ligase. After concentrating the sample and removing the ligation buffer, we reversed the crosslinks and deproteinated the sample in 50 μL cross-link reversal buffer (50mM Tris pH=8.0, 1% SDS, 0.25mM $CaCl_2$, and 0.5 mg per mL Proteinase K). We incubated the sample at 55°C to digest the histones, then increased the temperature to 68°C to reverse the crosslinks. After cross-link reversal, we separated the sample from the beads and purified on fresh SPRI beads at a ratio of 2:1[18]. DNA recovery was quantified by Qubit fluorometer. We prepared the sample for sequencing using the NEB Ultra library preparation kit according to the manufacturer's instructions, with one exception: prior to indexing PCR, the sample was enriched by pulldown on 30 μL Invitrogen C1 Streptavidin beads, then washed to remove non-biotinylated DNA fragments as follows: 1 wash with lithium wash buffer (1M LiCl, 0.5mM EDTA, 0.05% Tween-20), followed by 2 washes with sodium wash buffer (1M NaCl, 0.5mM EDTA, 0.05% Tween-20), followed by 2 washes with TE-Tween. We sent the library for sequencing at UC San Diego Institute for Genomic Medicine Genomics Center on a HiSeq 4000[19] (2x75bp).

We extracted genomic DNA from a whole blood sample from SC36 with the Qiagen Blood and Cell Culture Mini Kit, using 750 μL of starting material. We followed the protocol exactly, up

until the spooling step. The DNA was not sufficiently concentrated to precipitate, so we centrifuged the sample at 5000g for 15 minutes, washed it with cold 70% EtOH, and centrifuged it again at 5000g for 10 minutes. We removed the supernatant and air-dried the pellet. We resuspended the pellet in 50 μL of TE buffer on a shaker at 22°C overnight. We quantified DNA with a Qubit 2.0 dsDNA HS kit at 71.5 ng per μL (3.58 μg total). We verified the DNA size distribution with a pulse-field gel using a 0.75% agarose TAE gel, run at 75V for 16 hours, with the preset 5-150 kb program on the Pippin Pulse power Supply (version 1.3.2), and estimated the DNA fragments to range in size from 20-25 kb in length. We performed both a Rapid and a $1D^2$ Sequencing run (Oxford Nanopore, SQK-RAD002 and SQK-LSK308). For the Rapid library preparation, we used an input of 200 ng of DNA, as recommended by the manufacturer. We first treated the high molecular weight DNA with 2.5 μL of fragment repair mix and incubated for 1 minute at 30°C, followed by 1 minute at 75°C. We ligated the repaired DNA with Rapid Adapters using Blunt/TA Ligase (NEB). We quantified the Rapid libraries using a Qubit prior to sequencing. The Rapid sequencing libraries were run on an R9.5 flow cell using the NC_48hr_Sequencing_FLO-MIN107_SQK_RAD002 protocol. For the $1D^2$ libraries, we used the recommended 1 μg of high molecular weight DNA. We end-repaired and A-tailed the DNA using NEBNext UltraII End-Repair/dA-tailing mix (NEB). We cleaned up the end-repair product using AMPure XP beads and then ligated $1D^2$ adapters using Blunt/TA Ligase (NEB). We performed a subsequent ligation to add the Barcoded Adapter mix (BAM) using Blunt/TA Ligase (NEB). We quantified the $1D^2$ libraries using a Qubit prior to sequencing. The $1D^2$ libraries were run on an R9.5 flow cell using the NC_48hr_Sequencing_FLO-MIN107_SQK_LSK308 protocol. We base called all Fast5 raw reads generated from both sequencing runs using the latest Albacore software (Oxford Nanopore proprietary) (version 2.0.2).

We removed adapters from the eight Illumina libraries for SC36 with SeqPrep2 (https://github.com/jeizenga/SeqPrep2), using the default parameters except for increasing the quality score cutoff to 15 (-q 15) and reducing the minimum length of trimmed reads to 25 bp (-L 25). We used Trimmomatic[21] (version 0.33) to 1) remove additional Meyer Kircher IS3 adapters[16] using a seed mismatch of 2 and a simple clip threshold reduced to 5 for the shorter adapter sequence, 2) quality end trim using minimum qualities of 2 or 5 for leading and trailing ends of reads, respectively, 3) window quality trim, using a window size of 4 and a minimum quality of 15, and 4) remove reads shorter than 50 bp. We determined the expected genome assembly size by creating a histogram of kmer counts for k=45 with Jellyfish[22] (version 2.2.10), that we visualized with GenomeScope[23] to obtain a genome length estimate of 2.257Gb, which we used for calculating NG50 (Supplementary Table 1). We used this processed shotgun data to assemble a *de novo* genome using the Meraculous-2D Genome Assembler[24] (version 2.2.4), with diploid mode set to 1 and a kmer size of 45. The contig N50 of the shotgun assembly was 17.6 kb and the scaffold N50 was 36.6 kb. We had approximately 47X coverage of the genome from the eight Illumina shotgun libraries.

We scaffolded the Meraculous assembled genome using HiRise[18] (version 2.1.1) run in serial mode using the default parameters with the Chicago and Hi-C libraries as input. The resulting

scaffold N50 was 103.8 Mb. We further visualized the HiC assembly using HiGlass[25] after filtering for duplicates and valid Hi-C reads with pairtools (https://github.com/mirnylab/pairtools) (Supplementary Figure 1).

Given that the puma used for the SC36 shotgun assembly was a male, we had difficulty in assembling the X and Y chromosomes with Meraculous run in diploid mode 1. We identified potential X and Y scaffolds in the SC36 HiRise assembly by using Exonerate[26] (version 2.2.0) to align to known domestic cat X and Y chromosome genes[27]. We identified two scaffolds (scaffolds 2173 and 1964) which had numerous X chromosome mapping genes, and validated these scaffolds as being X chromosome scaffolds based on coverage of the Illumina shotgun data for SC36. We removed both scaffolds from the HiRise assembly. We identified one scaffold that confidently mapped exclusively to a Y chromosome gene, but were unable to validate it based on coverage, so we did not remove it from our HiRise assembly.

To generate an assembly for the X chromosome, we used shotgun data generated from a female puma from the Santa Monica Mountains, SMM13 (Section Additional Puma Sequencing), using Meraculous[24] (version 2.2.4) in diploid mode 1 and a kmer size of 45. We determined sex chromosome scaffolds in the SMM13 genome assembly using Exonerate[26] (version 2.2.0) by identifying scaffolds that align to known domestic cat X genes[27]. We identified three large scaffolds in this way, and validated them based on coverage for both SC36 and SMM13 using bedtools[28] (version 2.25.0) (Supplementary Figure 2). For SMM13, we found that these scaffolds had approximately the same coverage as the average genome-wide coverage. For SC36, we found these scaffolds had roughly half coverage of the average genome coverage. We added these three X chromosome scaffolds (scaffolds X1, X2, and X3) to the HiRise assembly for SC36.

We performed gap filling on the HiRise scaffolded assembly using Oxford Nanopore Technologies (ONT) long reads with the tool PBJelly, part of the PBSuite[29] (version 15.8.24). PBJelly also resolved the sizes of the gaps introduced by HiRise. We used Porechop (https://github.com/rrwick/Porechop) (version 0.2.3) to adapter trim the ONT reads. The resulting reads provided 1.2X fold coverage of the genome. We set PBJelly to correct only intra-scaffold gaps, and used the default minimum of 1 read spanning a gap. We reduced the number of gaps (represented in the assembly as a series of Ns) in the genome from 258,836 to 207,433 and reduced the total number of Ns in the genome from 154,284,192 to 132,359,239. Although our coverage from ONT was only 1.2X, the addition of these data to fill gaps between HiC-linked scaffolds recovered over 140 Mbp of sequence.

We used the genome improvement tool Pilon[30] (version 1.22) to correct sequence errors in the gaps that were filled with the high-error ONT data. We first aligned the Illumina shotgun data back to the PBJelly genome using bwa mem[31] (version 0.7.7) and marked duplicates with Picard toolkit MarkDuplicates (http://broadinstitute.github.io/picard) (version 1.114). We then ran Pilon with the alignment file as the "--frags" input, the PBJelly genome as the genome file, specified the genome as diploid, and used the default setting to fix all types of changes. We ran two iterations of alignment and consensus sequence calling.

We assessed this final version of the genome (PumCon1.0) by alignment to the domestic cat genome (GCA_000181335.4) using SyMAP[1] (version 4.2) using the BLAT default alignment and synteny parameters, but with Min Dots set to 10, and Top N set to 1 (Supplementary Figure 3). We also used the genome assessment tool BUSCO[12] (version 2.0.1) to evaluate genome completeness based on a set of conserved single-copy orthologous genes (human gene set; n=4104). In the PumCon1.0 genome, 93.0% of these genes are complete and present in a single copy only (Supplementary Table 2). The number of Illumina shotgun reads mapping to each version of the genome was calculated using the command "samtools view -q 30 -c" (Supplementary Table 1).

The final genome assembly was 2,432,985,507 bp in length and had an N50 of 100.53 Mb, with 178,994 gaps and 114,069,924 Ns. Ninety percent of the PumCon1.0 assembly is represented by 28 scaffolds, two of which are X related (scaffold_X1 and scaffold_X2). Thus 87.6% of the genome is represented on 26 autosomal scaffolds, each larger than 20 Mb.

We used a Trizol RNA extraction to obtain total RNA from whole blood collected from a wild puma (SC85) from the Santa Cruz Mountains. We removed unwanted globin mRNA by performing the GLOBINclear protocol (Thermo Fisher). For cDNA synthesis, we used 20 ng of input RNA, a reverse transcriptase (Clonetech), a SmartSeq2 template-switch oligo[32], and an Oligo-dT primer to enrich for poly-A+ RNA. We performed reverse transcription at 42°C for 1 hour. We treated the cDNA product with 1 μL of 1:10 dilution of RNase A (Thermo Fisher) and Lambda Exonuclease (NEB) and incubated at 37°C for 30 minutes. We amplified the cDNA for 18 cycles using KAPA Hifi Hotstart 2X readymix (KAPABiosystems) with an ISPCR primer[32]. We tagmented the amplified cDNA at 55°C for 7 minutes in a 20 μL reaction using a Tn5 enzyme to generate the RNA-Seq libraries. The Tn5 enzyme was loaded with custom oligos Tn5ME-A/R and Tn5ME-B/R[33]. We amplified the tagmented products for 13 cycles using KAPA Hifi (KAPA Biosystems) with Nextera primers. The amplified RNA-Seq library was size-selected targeting 300-800 bp using a 2% agarose E-gel (Thermo Fisher). The RNA-Seq library was then quantified using a Qubit and the size distribution was verified using an Agilent 2100 Bioanalyzer. We sent the RNA-Seq library for sequencing on a HiSeq 4000 at UC San Diego Institute for Genomic Medicine Genomics Center (2x100bp).

The PumCon1.0 genome was annotated by NCBI according to the NCBI Eukaryotic Genome Annotation Pipeline[34] using our cDNA library and a publicly available dataset generated from a wild puma from Arizona (SAMN02885420, SRX633288).

Additional puma genomes

We sampled 11 pumas for this study. Ten of these, including the individual used for the genome assembly (SC36), were used in a panel for analysis of demographic history and population structure. One was used to assemble the X chromosome. The nine additional pumas that formed our panel were: an additional puma from the Santa Cruz Mountains (SC29), a puma from Yellowstone National Park (YNP198), two pumas from the Big Cypress National Preserve that were members of the canonical (last remaining authentic) Florida panther population (CYP47, CYP51),

one puma from the Everglades National Park in Florida (EVG21), two pumas from the Santa Monica Mountains in Southern California (SMM12, SMM22), and two pumas from eastern Brazil (BR406, BR338). We also obtained a sample from a female puma from the Santa Monica Mountains for the purpose of assembling an X chromosome (SMM13) (Supplementary Table 3).

The Yellowstone puma was captured using specially-trained hounds, immobilized using a syringe dart (ketamine/medetomidine drug combination), and blood was obtained with aseptic techniques. Approval for Yellowstone sampling was granted by the National Park Service IACUC (#IMR_YELL_Stahler_Cougar_2015.A3). Capturing, handling and sampling of pumas from the Santa Cruz Mountains was approved by the IACUC at UCSC (protocol #Wilmc1101) and granted by the California Department of Fish and Wildlife[15]. Permission and approval to capture and handle pumas from the Santa Monica Mountains pumas were obtained from the National Park Service IACUC (PWR_SAMO_Riley.Sikich_MtnLion_2017) and the California Department of Fish and Wildlife[14]. Capture and sampling of Florida panthers were made possible by a cooperative agreement between the Florida Fish and Wildlife Conservation Commission and the US Fish and Wildlife Service (TE01553-3 and TE146761-1)[2]. Brazilian samples were collected by certified veterinarians following federal legislation and animal welfare guidelines. Sample BR406 was collected under federal permit SISBIO 40079-2; sample BR338 was collected under federal permits SISBIO 15024-3 and 19928-1, and used in a previous study of puma population genetics[13].

We aimed for high coverage (~30X), whole genome sequencing data for the ten pumas. We extracted DNA from blood samples using the Qiagen DNeasy Blood & Tissue kit using the same method as used for SC36. We prepared indexed Illumina libraries following the Meyer Kircher protocol[35]. We verified DNA concentrations and ~350-bp insert sizes by running the libraries on an Agilent 2200 TapeStation system.

We sent samples for sequencing at the National Genomics Infrastructure of SciLife in Stockholm, Sweden on an Illumina HiSeq XTen, Laboratório de Biotecnologia Animal at the Universidade de São Paulo in Brazil on an Illumina HiSeq 1500, and UC San Diego Institute for Genomic Medicine Genomics Center on an Illumina HiSeq 4000[19].

We also downloaded shotgun sequencing data for the African cheetah[36] (SRR2737512-SRR2737518) to use as the outgroup for our analyses. We selected cheetah data with an insert size of 170 bp and obtained a final coverage of 34X when mapped to PumCon1.0.

Variant Calling and Filtering

Prior to alignment of resequencing reads, we added the mitochondrial genome sequence for SC36 (Section Mitochondrial Genome Assemblies and Phylogeny) as a scaffold (scaffold_Mt) to the final nuclear sequence. Due to the high number of nuclear mitochondrial DNA segments (NUMTs) in felids[37], we sought to decrease erroneous mismappings of true mitochondrial DNA in the Illumina data to the NUMTs.

We removed adapters from all puma resequencing data and cheetah SRA data using SeqPrep2, discarding reads shorter than 25 bp in length. We then aligned reads to the PumCon1.0

genome, including the mitochondrial scaffold, using bwa mem[38] (version 0.7.12). We filtered alignments using samtools[39] (version 1.2.1) to keep alignments with a map quality score greater than or equal to 30, remove secondary alignments, and keep reads where both reads in the read pair mapped. Within each library, we removed duplicate sequences due to PCR amplification using samtools rmdup[39] (version 0.1.18). Realignment around insertions and deletions was performed using GATK Realigner Target Creator and Indel Realignment[40] (version 3.5.0). We called variants in each sample using GATK HaplotypeCaller[40] (version 3.7.0), with a minimum base quality score of 18, and emitting all sites, including invariant positions.

We generated three sets of genotypes: two sets consisted of the ten pumas (on was LD filtered and the other was not LD filtered) and the third included the ten pumas plus the cheetah for use as the outgroup. For the puma only data set, we joint genotyped all ten puma samples using GATK GenotypeGVCFs[40] (version 3.7.0), including non-variant sites. For the data set including the African cheetah, we again used GATK GenotypeGVCFs to perform joint genotyping for all 11 samples, but emitted only variant sites.

For all variant files, we masked or removed sites that were not biallelic SNPs and then filtered biallelic SNPs. We determined filtering thresholds by visualizing parameter distributions. We used GATK VariantFiltration and SelectVariants[40] to filter strand odds ratio (SOR > 3.0), Fisher strand bias (FS > 60.00), quality by depth (QD < 2.00), mapping quality (MQ < 40.00 and MQRankSum < -10.00), read position (-8.00 < ReadPosRankSum > 8.00), and excess heterozygosity in accordance with Hardy-Weinberg equilibrium to remove potentially paralogous genomic regions (ExcessHet > 10.0). We used GATK VariantFiltration and SelectVariants[40] to remove variants above a cumulative depth threshold (DP > 1500 for the puma only variant file, DP > 1650 for the puma and cheetah variant file). We then used vcftools[41] (version 0.1.12b) to remove singleton variants where only one individual carried one copy of a different allele (--mac 2), sites where any individual had a depth less than 10 (--minDP 10.0), and sites where any of the ten pumas did not have a base called (--max-missing-count 0). We only used autosomal scaffolds for further analyses, removing mitochondrial and X chromosome related scaffolds (scaffold Mt, X1, X2, X3, 869, 1862). Scaffolds 869 and 1862 were removed due to syntenic mappings with the X chromosome of FelisCatus9.0 in SyMAP[1]. For the LD filtered variant files, we performed LD filtering using PLINK[42] (version 1.90b4.4) with the command "--indep-pairwise 100 10 0.25".

The non-LD filtered puma only variant file contained 8,212,535 SNPs. The final LD-filtered puma only variant file contained 166,037 SNPs. The puma and cheetah variant LD-filtered variant file contained 557,741 SNPs. The larger number of variants in the puma and cheetah file is due to the high numbers of sites where the cheetah carries two of the alternate allele, while all pumas carry the reference allele.

Using the non-LD filtered SNP calls from the puma only data set, we generated a fasta file for each sample using GATK FastaAlternateReferenceMaker[40] (version 3.7.0), with heterozygous positions represented by IUPAC codes. Failed SNP sites (see filters above) and failed individual genotypes (RGQ<20, depth<10) were masked to Ns using the *maskfasta* function in bedtools[28].

Mitochondrial genome assemblies and phylogeny inference

We used Unicycler[43] (version 0.4.4) in hybrid assembly mode to assemble an initial mitochondrial sequence for SC36. To decrease our initial input dataset, we first identified mitochondrial mapping reads. To do this, we mapped adapter-trimmed Illumina and ONT reads from SC36 to a publicly available puma reference mitochondrial sequence (KP202261.1) using bwa mem[31] (version 0.7.12) and bwa mem ont2d, respectively. We took the reads in the Illumina and ONT alignment files that mapped to the reference mitochondrial sequence and converted them into fastq format using bedtools[44] bamToFastq. We used both the Illumina and ONT mitochondrial-mapped readsets as input into Unicycler as long reads using the flag "l" and unpaired reads using the flag "-s", respectively. The assembly created with Unicycler was 17,065 bp in length, circular, and had a depth of 490X. To validate this assembly, we used the Unicycler output as the reference sequence in an assembly using mapping iterative assembler (mia)[45] (version 1.0) using roughly 20 million randomly selected adapter-trimmed Illumina shotgun reads (mia flags: -i -c -C -F -k 13). The resulting mia assembly was identical in sequence to the inputted Unicycler assembly, and thus was used as the final mitochondrial sequence for SC36.

We used adapter-trimmed Illumina shotgun data to assemble the mitochondrial genome sequences of the remaining nine pumas with mia, using the SC36 mitochondrial sequence as the reference. The coverages of these mitochondrial assemblies ranged from 35X to 138X. For each of the nine assemblies, we filtered using a consensus threshold of 90% and required 10X coverage per site. Any site which did not meet these requirements were changed to an 'N', resulting in between 2 and 86 Ns per assembly. We removed two repetitive sequences located in the control region, RS2 (sites 16,511-16,861) and RS3 (sites 279-657), from each of the mitochondrial sequences. These regions are known to have highly variable numbers of repeats[46], and were difficult to accurately resolve using short read data. We annotated the mitochondrial genomes using MITOS[47].

We ran PartitionFinder[48] (version 1.1.1) and jModelTest2[49] (version 2.1.6) to determine the partitions and best substitution model. Due to the small number of substitutions seen in the pumas, PartitionFinder suggested a single mitochondrial partition. jModelTest2 recommended a GTR+GAMMA substitution model for tree building.

We used muscle[50] (version 3.8.31) to align our ten assembled puma mitochondrial genomes, the publicly available puma reference mitochondrial sequence (KP202261.1), and a cheetah mitochondrial sequence (KP202271.1) as the outgroup. We used RAxML[51] (version 8.2.4) to produce a maximum likelihood phylogeny, with a GTR+GAMMA evolutionary model, running one hundred bootstrap replicates. We also ran tree inference including the RS2 and/or RS3 repetitive regions, and saw little change in the bootstrap support values and no change to the topology of the tree.

We estimated divergence times between South and North American pumas using a prior composite estimate of the feline mitochondrial divergence rate of 1.15%bp per million years[52,53]. Branch lengths were taken from a phylogeny that did not include RS2 or RS3, and were used to

calculate divergence dates using the formula $\sigma = 2\lambda T$, where σ is percent divergence between a pair of sequences, λ is the rate of mitochondrial divergence, and T is time[53].

Demographic history

We used the pairwise sequentially Markovian coalescent (PSMC) model[3] to estimate the historical effective population size of puma populations. Of note to our sampled population histories, is that the Brazil pumas used in this analysis originate from eastern South America, which prior work has shown is the centrum of present-day puma diversity[52], and likely represents the most ancestral puma lineage. The input was a realigned alignment file of 26 autosomal scaffolds larger than 20 Mb, excluding sex chromosome associated scaffolds (Section Variant calling and filtering). We filtered the alignment file for each puma to include sites which had between one third and twice the average coverage for that puma. We used a generation time of 5 years and a mutation rate of 0.5e-8 per bp per generation, based on previous estimates of the feline mutation rate[4]. We performed one hundred replicate bootstraps for each individual per the software instructions (Supplementary Figure 5).

We also ran the PSMC tool on regions of the alignment file which were identified as outbred based on our ROH hidden Markov model (Section Runs of Homozygosity). We masked regions of homozygosity for each of the pumas, and re-ran the PSMC analysis to compare the demographic estimates. We observed minimal differences between the two sets (Supplementary Figure 6).

We investigated the divergence time between our male pumas by running PSMC modeling of pseudo-diploid sequences using pairs of haploid male X chromosomes to avoid the need to phase the data. In this way, we can approximate the population split time between two populations, as the y-axis in the PSMC plot can be thought of as the inverse of the rate of coalescence at a time along the x-axis[54]. We used an alignment file containing the three largest X scaffolds (scaffold_X1, scaffold_X2, and scaffold_X3), for a total of ~103 Mbp of the X chromosome sequence. We filtered the input to only include sites which had up to the average coverage of the genome for each puma. We created a pseudohaploid fasta file by randomly selecting a single high quality base using samtools (mpileup -Q 40 -q 30)[55] and pu2fa (https://github.com/Paleogenomics/Chrom-Compare). We combined the pseudohaploid fasta file of each male North American puma with that of either of the two Brazilian pumas, and ran PSMC as above (Supplementary Figure 7). To account for the lower mutation rate on the X than the autosomes, we used a formula[3] to convert the autosomal mutation rate into the X mutation rate, using a male to female mutation ratio of 1.4 from the domestic cat[5]. This provided a per generation mutation rate for the X chromosome of 0.472e-8 per bp per generation, using a generation time of 5 years.

Population structure

We used SmartPCA from the EIGENSOFT[56] (version 6.1.4) package to run principal component analysis on the LD-filtered variant file for the ten pumas, which consisted of 166,037

SNPs. We converted the variant file into eigenstrat format using PGDSpider2[57] (version 2.1.0.0) for input into SmartPCA.

We constructed a tree to show population splits using Treemix[6], both with and without the admixed sample EVG21. We used the LD-filtered variant file with the ten pumas and the cheetah as input, which consisted of 557,74 SNPs, and ran Treemix by grouping 5,000 SNPs into one window (k). For the dataset including EVG21, the tree with the highest log likelihood predicted one migration, and explained 99.91% of the variation. In the dataset without EVG21, the best model predicted no migrations and explained 99.91% of the variation.

We used the software STRUCTURE[8] (version 2.3.4) to infer the population structure of the pumas. We converted the LD filtered variant file with 10 pumas and 166,037 SNPs into the input format using PGDSpider2[57] (version 2.1.0.0). We ran 10 replicates of STRUCTURE for values from one to ten for the number of populations (K), using an admixture model with a degree of admixture (alpha) value of 1 for each K, 10,000 burns ins, and running 10,000 MCMC repetitions. We used the software CLUster Matching and Permutation Program (CLUMPP)[9] (version 1.1.2) under the greedy algorithm to align the ten replicates for each K into one representative mean output matrix for plotting (Supplementary Figure 11). We identified the best K based on mean likelihood L(K) and delta K values[7]. For our dataset, K=3 had the largest delta K value, and showed a break in slope for mean L(K) as well (Supplementary Figure 10). Although our analyses indicate that K=3 best explains our panel, due to our sampling scheme, we are unable to determine the true number of clusters for the species overall. We do note that all population level analyses performed indicate that our samples are best described by three clusters.

Genome-wide heterozygosity

We calculated the average coverage of each puma using samtools depth on the realigned alignment file, removing scaffolds for the mitochondria and X chromosome (scaffolds Mt, X1, X2, X3, 869, 1862). We calculated genome-wide heterozygosity by generating a pileup file from map quality and base quality filtered alignment files using samtools[55] (version 1.3.1) (mpileup -q 30 -Q 30) at all sites in the genome with exactly the average coverage depth for that individual. We created a histogram by binning the sites based on the number of reads representing the reference allele. We visually classified which bins were designated as homozygous or heterozygous. We calculated the genome-wide heterozygosity by summing all heterozygous bins and dividing by the total number of genome-wide sites used in the analysis.

Additionally, we examined sliding window heterozygosity using custom script that counts heterozygous positions in the IUPAC coded fasta files. We estimated average heterozygosity in 100-kb windows and used the outputted counts of the number of heterozygous positions and the number of positions with a genotype call to obtain another estimate of average genome-wide heterozygosity (Supplementary Table 4). The slightly higher values observed for the IUPAC estimates could be due to the pileup method being a more conservative method for calling a site heterozygous than GATK FastaAlternateReferenceMaker, which was used with the IUPAC method.

Lastly, we calculated genome heterozygosity in a non-reference based method so as to prevent any effects of genome quality on the estimations (Supplementary Table 4). We used adapter-trimmed shotgun data for each of the ten pumas to obtain kmer frequency distributions for k=21 for each puma using Jellyfish[22] (version 2.2.10) and visualized the results using GenomeScope[23].

Runs of homozygosity

We used a hidden Markov model (HMM) to identify ROH by identifying transitions between inbred and outbred regions of the genome (https://github.com/russcd/Heterozygosity_HMM). We first estimated HMM model parameters from the data (Supplementary Table 5). For each of the eight male pumas, we used the sample's heterozygosity estimate from the two large X scaffolds as an estimate of the genotyping error rate. For the two female pumas, we used the estimate from another sample that was geographically close by and had similar sequencing coverage. For each sample, we then estimated the rate of heterozygosity in outbred regions by visually selecting clearly outbred regions and determining the mean heterozygosity across those regions. We determined a single transition rate parameter (t=1e-50) by running different transition parameters and visually inspecting the results. We used these parameters and the filtered fasta files with IUPAC codes as input into the ROH HMM program. For downstream analyses, we used only the 26 largest autosomal scaffolds and discarded ROH less than 2 Mb. We converted the ROH tract lengths to generations using an estimated average recombination rate from the domestic cat of 1.1 cM per Mb[58] and the equation $g = 100/(2rL)$, where g is the time in generations, r is the recombination rate, and L is the length of the ROH tract in Mb[59].

We also used the sliding window approach in PLINK[42] (version 1.90b4.4) to identify ROH for comparison with our ROH HMM. We used the non-LD filtered puma variant file as input. We relaxed the parameters (--homozyg-window-het 20 --homozyg-window-missing 20 --homozyg-window-threshold 0.02 --homozyg-het 750 --homozyg-kb 500) to prevent sequencing errors from breaking up homozygous tracts. Even with the relaxed parameters, PLINK still tended to break up long tracts (Supplementary Figure 14). Since accurate estimates of tract lengths were key to our inbreeding analysis, we used the ROH called by our ROH HMM program for further analyses.

The low frequency of short ROH observed in the genome of the admixed Everglades panther (EVG21) relative to the other Florida panthers led us to believe that admixture in previous generations had prevented the formation of short ROH because an individual cannot have a shared maternal and paternal ancestor that dates to before the admixture event. To test our hypothesis, we used Ancestry_HMM[10,11] to classify tracts of ancestry in the IUPAC coded fasta file of EVG21 into three types: pure Central/South American ancestry, pure Floridian ancestry, and mixed Central/South American and Floridian ancestry. We used the two Brazil samples (BR338 and BR406) as proxies for Central/South American ancestry, and the two Big Cypress samples (CYP47

and CYP51) as proxies for Floridian ancestry. Because small sample sizes preclude accurate estimates of LD and because the program is sensitive to sites in strong LD, we pruned all ancestry informative markers within 250 kb of another site. The genome of EVG21 was composed of 21.98% Central/South American ancestry, 28.24% Floridian ancestry, and 49.58% mixed ancestry based on the HMM (Supplementary Figure 15). Using ROH greater than 2 Mb that we identified with the ROH HMM, we classified each ROH as one of the three ancestry types. We did this by using the *intersect* function in bedtools[44] (version 2.25.0) with the parameters "-e -f 0.90" such that 90% of any ROH identified was only one ancestry type. The results of this analysis classified all ROH as either pure Florida or pure Central/South American ancestry. We saw no ROH that were classified as being of mixed ancestry. Thus, admixture effectively prevents the formation of mixed ancestry ROH (Supplementary Figures 15, 16). ROH make up 76% of the Central/South American ancestry regions of the genome, 63% of the Floridian ancestry regions of the genome, and 0% of the mixed ancestry regions of the genome. We tested for significant differences in the number of ROH identified per ancestry type using an exact test of the ratio in a Poisson distribution. We found that significantly more ROH were of Floridian ancestry relative to mixed ancestry (p=7.276e-12), and significantly more ROH were of Central/South American ancestry than of mixed ancestry (p=1.526e-05). Additionally, significantly more ROH were of Floridian ancestry than Central/South American ancestry (p=0.006456). The identification of a greater number of ROH of Floridian ancestry, especially ROH of shorter length (Supplementary Figure 16), is consistent with the longer history of sustained inbreeding in the Floridian population.

We next estimated the proportion of the ROH that are shared between pairs of pumas. First, we used the *intersect* function in bedtools[44] to find genomic regions where ROH overlap between pairs of samples. Then we used a custom script to generate a hybrid diploid fasta file from the IUPAC coded fasta files for each pair of samples. The script first makes a pseudo-haploidized sequence for each sample by randomly selecting one of the two bases at heterozygous sites. The script then uses a pair of pseudo-haploidized sequences to generate a hybrid diploid fasta file by using IUPAC codes to represent differing bases between the two samples, while shared bases between the two samples are represented by the base itself. We ran the ROH HMM program on the generated fasta file, using the same transition rate parameter as above (t=1e-50) and the outbred heterozygosity rate and genotyping error rate averaged across all ten pumas (h=0.00135, e=0.0000765). These ROH indicate where two individuals share regions of the genome IBD. To determine if ROH that overlap between two samples were also IBD, we used the *intersect* function in bedtools[44] (version 2.25.0) to find the intersection between the ROH that overlap between the two samples and the ROH generated from the hybrid diploid fasta file. Finally, from these outputs, for each pair of pumas we calculated the proportion of the genome that occurs in ROH that are IBD (Supplementary Table 6).

References

1. Soderlund, C., Nelson, W., Shoemaker, A. & Paterson, A. SyMAP: A system for discovering and viewing syntenic regions of FPC maps. *Genome Res.* **16**, 1159–1168 (2006).
2. Johnson, W. E. *et al.* Genetic restoration of the Florida panther. *Science* **329**, 1641–1645 (2010).
3. Li, H. & Durbin, R. Inference of human population history from individual whole-genome sequences. *Nature* **475**, 493–496 (2011).
4. Cho, Y. S. *et al.* The tiger genome and comparative analysis with lion and snow leopard genomes. *Nat. Commun.* **4**, 2433 (2013).
5. Sayres, M. A. W., Wilson Sayres, M. A., Venditti, C., Pagel, M. & Makova, K. D. Do variations in substitution rates and male mutation bias correlate with life-history traits? A study of 32 mammalian genomes. *Evolution* **65**, 2800–2815 (2011).
6. Pickrell, J. K. & Pritchard, J. K. Inference of population splits and mixtures from genome-wide allele frequency data. *PLoS Genet.* **8**, e1002967 (2012).
7. Evanno, G., Regnaut, S. & Goudet, J. Detecting the number of clusters of individuals using the software STRUCTURE: a simulation study. *Mol. Ecol.* **14**, 2611–2620 (2005).
8. Falush, D., Stephens, M. & Pritchard, J. K. Inference of population structure using multilocus genotype data: linked loci and correlated allele frequencies. *Genetics* **164**, 1567–1587 (2003).
9. Jakobsson, M. & Rosenberg, N. A. CLUMPP: a cluster matching and permutation program for dealing with label switching and multimodality in analysis of population structure. *Bioinformatics* **23**, 1801–1806 (2007).
10. Corbett-Detig, R. & Nielsen, R. A Hidden Markov Model Approach for Simultaneously

Estimating Local Ancestry and Admixture Time Using Next Generation Sequence Data in Samples of Arbitrary Ploidy. *PLoS Genet.* **13**, e1006529 (2017).

11. Medina, P., Thornlow, B., Nielsen, R. & Corbett-Detig, R. Estimating the Timing of Multiple Admixture Pulses During Local Ancestry Inference. *Genetics* **210**, 1089–1107 (2018).
12. Simão, F. A., Waterhouse, R. M., Ioannidis, P., Kriventseva, E. V. & Zdobnov, E. M. BUSCO: assessing genome assembly and annotation completeness with single-copy orthologs. *Bioinformatics* **31**, 3210–3212 (2015).
13. Matte, E. M. *et al.* Molecular evidence for a recent demographic expansion in the puma (Puma concolor) (Mammalia, Felidae). *Genet. Mol. Biol.* **36**, 586–597 (2013).
14. Riley, S. P. D. *et al.* Individual behaviors dominate the dynamics of an urban mountain lion population isolated by roads. *Curr. Biol.* **24**, 1989–1994 (2014).
15. Wilmers, C. C. *et al.* Scale dependent behavioral responses to human development by a large predator, the puma. *PLoS One* **8**, e60590 (2013).
16. Meyer, M. & Kircher, M. Illumina sequencing library preparation for highly multiplexed target capture and sequencing. *Cold Spring Harb. Protoc.* **2010**, (2010).
17. Deng, X. *et al.* Bipartite structure of the inactive mouse X chromosome. *Genome Biol.* **16**, 152 (2015).
18. Putnam, N. H. *et al.* Chromosome-scale shotgun assembly using an in vitro method for long-range linkage. *Genome Res.* **26**, 342–350 (2016).
19. Bentley, D. R. *et al.* Accurate whole human genome sequencing using reversible terminator chemistry. *Nature* **456**, 53–59 (2008).
20. Lieberman-Aiden, E. *et al.* Comprehensive mapping of long-range interactions reveals folding

principles of the human genome. *Science* **326**, 289–293 (2009).

21. Bolger, A. M., Lohse, M. & Usadel, B. Trimmomatic: a flexible trimmer for Illumina sequence data. *Bioinformatics* **30**, 2114–2120 (2014).

22. Marçais, G. & Kingsford, C. A fast, lock-free approach for efficient parallel counting of occurrences of k-mers. *Bioinformatics* **27**, 764–770 (2011).

23. Vurture, G. W. *et al.* GenomeScope: fast reference-free genome profiling from short reads. *Bioinformatics* **33**, 2202–2204 (2017).

24. Chapman, J. A. *et al.* Meraculous: de novo genome assembly with short paired-end reads. *PLoS One* **6**, e23501 (2011).

25. Kerpedjiev, P. *et al.* HiGlass: web-based visual exploration and analysis of genome interaction maps. *Genome Biol.* **19**, 125 (2018).

26. Slater, G. S. C. & Birney, E. Automated generation of heuristics for biological sequence comparison. *BMC Bioinformatics* **6**, 31 (2005).

27. Pearks Wilkerson, A. J. *et al.* Gene discovery and comparative analysis of X-degenerate genes from the domestic cat Y chromosome. *Genomics* **92**, 329–338 (2008).

28. Quinlan, A. R. & Hall, I. M. BEDTools: a flexible suite of utilities for comparing genomic features. *Bioinformatics* **26**, 841–842 (2010).

29. English, A. C. *et al.* Mind the gap: upgrading genomes with Pacific Biosciences RS long-read sequencing technology. *PLoS One* **7**, e47768 (2012).

30. Walker, B. J. *et al.* Pilon: an integrated tool for comprehensive microbial variant detection and genome assembly improvement. *PLoS One* **9**, e112963 (2014).

31. Li, H. Aligning sequence reads, clone sequences and assembly contigs with BWA-MEM.

ARXIV (03/2013). doi:2013arXiv1303.3997L

32. Picelli, S. *et al.* Smart-seq2 for sensitive full-length transcriptome profiling in single cells. *Nat. Methods* **10**, 1096–1098 (2013).

33. Picelli, S. *et al.* Full-length RNA-seq from single cells using Smart-seq2. *Nat. Protoc.* **9**, 171–181 (2014).

34. Françoise, T.-N. P., Alexander, S. P., Terence, M. P. & Dicuccio Md Michael And. *Eukaryotic Genome Annotation Pipeline*. (National Center for Biotechnology Information (US), 2013).

35. Meyer, M. & Kircher, M. Illumina sequencing library preparation for highly multiplexed target capture and sequencing. *Cold Spring Harb. Protoc.* **2010**, (2010).

36. Dobrynin, P. *et al.* Genomic legacy of the African cheetah, Acinonyx jubatus. *Genome Biol.* **16**, 277 (2015).

37. Lopez, J. V., Yuhki, N., Masuda, R., Modi, W. & O'Brien, S. J. Numt, a recent transfer and tandem amplification of mitochondrial DNA to the nuclear genome of the domestic cat. *J. Mol. Evol.* **39**, 174–190 (1994).

38. Li, H. & Durbin, R. Fast and accurate short read alignment with Burrows-Wheeler transform. *Bioinformatics* **25**, 1754–1760 (2009).

39. Li, H. *et al.* The Sequence Alignment/Map format and SAMtools. *Bioinformatics* **25**, 2078–2079 (2009).

40. McKenna, A. *et al.* The Genome Analysis Toolkit: a MapReduce framework for analyzing next-generation DNA sequencing data. *Genome Res.* **20**, 1297–1303 (2010).

41. Danecek, P. *et al.* The variant call format and VCFtools. *Bioinformatics* **27**, 2156–2158 (2011).

42. Chang, C. C. *et al.* Second-generation PLINK: rising to the challenge of larger and richer

datasets. *Gigascience* **4**, (2015).

43. Wick, R. R., Judd, L. M., Gorrie, C. L. & Holt, K. E. Unicycler: Resolving bacterial genome assemblies from short and long sequencing reads. *PLoS Comput. Biol.* **13**, e1005595 (2017).

44. Quinlan, A. R. & Hall, I. M. BEDTools: a flexible suite of utilities for comparing genomic features. *Bioinformatics* **26**, 841–842 (2010).

45. Green, R. E. *et al.* A complete Neandertal mitochondrial genome sequence determined by high-throughput sequencing. *Cell* **134**, 416–426 (2008).

46. Lopez, J. V., Yuhki, N., Masuda, R., Modi, W. & O'Brien, S. J. Numt, a recent transfer and tandem amplification of mitochondrial DNA to the nuclear genome of the domestic cat. *J. Mol. Evol.* **39**, 174–190 (1994).

47. Bernt, M. *et al.* MITOS: improved de novo metazoan mitochondrial genome annotation. *Mol. Phylogenet. Evol.* **69**, 313–319 (2013).

48. Lanfear, R., Calcott, B., Ho, S. Y. W. & Guindon, S. Partitionfinder: combined selection of partitioning schemes and substitution models for phylogenetic analyses. *Mol. Biol. Evol.* **29**, 1695–1701 (2012).

49. Darriba, D., Taboada, G. L., Doallo, R. & Posada, D. jModelTest 2: more models, new heuristics and parallel computing. *Nat. Methods* **9**, 772 (2012).

50. Edgar, R. C. MUSCLE: multiple sequence alignment with high accuracy and high throughput. *Nucleic Acids Res.* **32**, 1792–1797 (2004).

51. Stamatakis, A. RAxML version 8: a tool for phylogenetic analysis and post-analysis of large phylogenies. *Bioinformatics* **30**, 1312–1313 (2014).

52. Culver, M., Johnson, W. E., Pecon-Slattery, J. & O'Brien, S. J. Genomic ancestry of the

American puma (Puma concolor). *J. Hered.* **91**, 186–197 (2000).

53. Lopez, J. V., Culver, M., Stephens, J. C., Johnson, W. E. & O'Brien, S. J. Rates of nuclear and cytoplasmic mitochondrial DNA sequence divergence in mammals. *Mol. Biol. Evol.* **14**, 277–286 (1997).

54. Mazet, O., Rodríguez, W., Grusea, S., Boitard, S. & Chikhi, L. On the importance of being structured: instantaneous coalescence rates and human evolution--lessons for ancestral population size inference? *Heredity* **116**, 362–371 (2016).

55. Li, H. *et al.* The Sequence Alignment/Map format and SAMtools. *Bioinformatics* **25**, 2078–2079 (2009).

56. Patterson, N., Price, A. L. & Reich, D. Population Structure and Eigenanalysis. *PLoS Genet.* **2**, e190 (2006).

57. Lischer, H. E. L. & Excoffier, L. PGDSpider: an automated data conversion tool for connecting population genetics and genomics programs. *Bioinformatics* **28**, 298–299 (2012).

58. Dumont, B. L. & Payseur, B. A. Evolution of the genomic rate of recombination in mammals. *Evolution* **62**, 276–294 (2008).

59. Kardos, M. *et al.* Genomic consequences of intensive inbreeding in an isolated wolf population. *Nat Ecol Evol* **2**, 124–131 (2018).
