# Supplementary material for: Puma genomes from North and South America provide insights into the genomic consequences of inbreeding
Source: Nat Commun. 2019 Oct 18;10:4769. doi: 10.1038/s41467-019-12741-1 (PMC6800433; doi:10.1038/s41467-019-12741-1)
Supplement: Supplementary file 3 — Reporting Summary [file 41467_2019_12741_MOESM3_ESM.pdf]

nature research

Corresponding author(s): Beth Shapiro

Last updated by author(s): 09/17/2019

Reporting Summary

Nature Research wishes to improve the reproducibility of the work that we publish. This form provides structure for consistency and transparency in reporting. For further information on Nature Research policies, see Authors & Referees and the Editorial Policy Checklist.

Statistics

For all statistical analyses, confirm that the following items are present in the figure legend, table legend, main text, or Methods section.

n/a	Confirmed	
☐	☒	The exact sample size (*n*) for each experimental group/condition, given as a discrete number and unit of measurement
☐	☒	A statement on whether measurements were taken from distinct samples or whether the same sample was measured repeatedly
☐	☒	The statistical test(s) used AND whether they are one- or two-sided<br>*Only common tests should be described solely by name; describe more complex techniques in the Methods section.*
☒	☐	A description of all covariates tested
☒	☐	A description of any assumptions or corrections, such as tests of normality and adjustment for multiple comparisons
☒	☐	A full description of the statistical parameters including central tendency (e.g. means) or other basic estimates (e.g. regression coefficient) AND variation (e.g. standard deviation) or associated estimates of uncertainty (e.g. confidence intervals)
☐	☒	For null hypothesis testing, the test statistic (e.g. *F*, *t*, *r*) with confidence intervals, effect sizes, degrees of freedom and *P* value noted<br>*Give P values as exact values whenever suitable.*
☐	☒	For Bayesian analysis, information on the choice of priors and Markov chain Monte Carlo settings
☒	☐	For hierarchical and complex designs, identification of the appropriate level for tests and full reporting of outcomes
☒	☐	Estimates of effect sizes (e.g. Cohen's *d*, Pearson's *r*), indicating how they were calculated

Our web collection on statistics for biologists contains articles on many of the points above.

Software and code

Policy information about availability of computer code

Data collection	No software was used for data collection
Data analysis	SepPrep2, Trimmomatic v0.33, Meraculous v2.2.4, HiRise v2.1.1, Exonerate v2.2.0, PBJelly v15.8.24, Porechop v0.2.3, Pilon v1.22, bwa mem v0.7.7, Picard toolkit v1.114, SyMap v4.2,BUSCO v2.0.1, samtools v1.2.1, samtools v0.1.18, GATK v3.5.0, GATK v3.7.0, PLINK v1.90b4.4, Unicycler v 0.4.4, bedtools v2.25.0, mia v1.0, PartitionFinder v1.1.1, jModelTest v2.1.6, RaxML v8.2.4, PSMC, EIGENSOFT v6.1.4, PGDSpider2 v2.1.0.0, Treemix v1.13, STRUCTURE v2.3.4, CLUMPP v1.1.2, GenomeScope, ROH_HMM, Ancestry_HMM

For manuscripts utilizing custom algorithms or software that are central to the research but not yet described in published literature, software must be made available to editors/reviewers. We strongly encourage code deposition in a community repository (e.g. GitHub). See the Nature Research guidelines for submitting code & software for further information.

Data

Policy information about availability of data

All manuscripts must include a data availability statement. This statement should provide the following information, where applicable:

- Accession codes, unique identifiers, or web links for publicly available datasets
- A list of figures that have associated raw data
- A description of any restrictions on data availability

Shotgun data used for the genome assembly have been deposited in the SRA under accession IDs SRR7148342-SRR7148354. The PumCon1.0 genome is available on GenBank under GCF_003327715. The RNA-Seq data is publicly available on the SRA under the ID SRX4067841. Reads for the panel of mountain lions have been deposited in the SRA under the accession IDs SRR7639695-6, SRR7542886-8, SRR7660678-9, SRR7664677-8, SRR7956993-4, SRR7610940-1, SRR7661934-5, SRR7690239-40, SRR7543017-8, SRR7537344-5, and SRR7148342-54. The annotated mitochondrial assemblies for the ten mountain lion are available on GenBank under accession numbers MH807447, MH814703-MH814707, and MH818219-MH818222.

Field-specific reporting

Please select the one below that is the best fit for your research. If you are not sure, read the appropriate sections before making your selection.

☐ Life sciences ☐ Behavioural & social sciences ☒ Ecological, evolutionary & environmental sciences

For a reference copy of the document with all sections, see nature.com/documents/nr-reporting-summary-flat.pdf

Ecological, evolutionary & environmental sciences study design

All studies must disclose on these points even when the disclosure is negative.

Study description	Genomic study of pumas from North and South America
Research sample	We selected pumas from populations across North and South America, with greater focus on sampling the diversity of North America. We were able to obtain samples with known pedigree information (Florida and Santa Monica). All samples were collected from adult pumas.
Sampling strategy	Since our study's goal was to obtain high coverage genomes from multiple individuals, we were limited in the number of individuals we could sequence. We thus aimed to obtain representatives from populations of interest, attempting to have more than one representative per population.
Data collection	Puma samples were collected by a number of groups, and blood samples were used as input for the creation of genomic datasets.
Timing and spatial scale	All puma samples are for the present time point, and were collected from the 1990s (Florida) to 2015 (Santa Cruz)
Data exclusions	No data was excluded from this study
Reproducibility	We did no specific experiments for reproducibility, but we believe sampling individuals from the same populations provides evidence of experimental replicability
Randomization	Samples were allocated into groups based on field observations, prior genetic testing from earlier research, and geographic origins. All analyses supported geographical clustering for the pumas used in this study.
Blinding	No blinding was used in this study

Did the study involve field work? ☒ Yes ☐ No

Field work, collection and transport

Field conditions	Samples were collected year round in locations in North and South America.
Location	Sampling was performed in Yellowstone Park in Montana, the Santa Cruz Mountains in northern California, the Santa Monica Mountains in southern California, southern Florida, and Minas Gerais state and Sao Paolo state in Brazil.
Access and import/export	Permits and approval for capture and sampling of pumas used in this study were obtained from the appropriate governing body. Approval bodies: National Park Service IACUC, IACUC at UC Santa Cruz, California Department of Fish and Wildlife, state and federal agencies in Brazil, and US Fish and Wildlife Service
Disturbance	Hounds used for capture are removed from the location once anesthesia is administered to the puma to reduce stress. Vital signs are monitored and addressed accordingly to ensure the safety and health of the animal.

Reporting for specific materials, systems and methods

We require information from authors about some types of materials, experimental systems and methods used in many studies. Here, indicate whether each material, system or method listed is relevant to your study. If you are not sure if a list item applies to your research, read the appropriate section before selecting a response.

Materials & experimental systems

n/a	Involved in the study
☒	☐ Antibodies
☒	☐ Eukaryotic cell lines
☒	☐ Palaeontology
☐	☒ Animals and other organisms
☒	☐ Human research participants
☒	☐ Clinical data

Methods

n/a	Involved in the study
☒	☐ ChIP-seq
☒	☐ Flow cytometry
☒	☐ MRI-based neuroimaging

Animals and other organisms

Policy information about studies involving animals; ARRIVE guidelines recommended for reporting animal research

Laboratory animals	This study did not involve laboratory animals
Wild animals	This study involved temporary captures of wild pumas to extract blood samples. All capturing and sampling was approved by the appropriate IACUC as detailed in the methods.
Field-collected samples	Field collected blood samples were kept in -80C freezers until used for laboratory analyses
Ethics oversight	Permits and approval for capture and sampling of pumas used in this study were obtained from the National Park Service IACUC, IACUC at UC Santa Cruz, California Department of Fish and Wildlife, state and federal agencies in Brazil, and US Fish and Wildlife Service

Note that full information on the approval of the study protocol must also be provided in the manuscript.
